# DYRK1A inhibitors leucettines and TGF-β inhibitor additively stimulate insulin production in beta cells, organoids, and isolated mouse islets

**DOI:** 10.1371/journal.pone.0285208

**Published:** 2023-05-17

**Authors:** Barbara Pucelik, Agata Barzowska, Anna Czarna

**Affiliations:** 1 Malopolska Centre of Biotechnology, Jagiellonian University, Gronostajowa, Krakow, Poland; 2 Doctoral School of Exact and Natural Sciences, Jagiellonian University, Krakow, Poland; Universidade Federal de Minas Gerais, BRAZIL

## Abstract

The decreased β-cell mass and impaired β-cell functionality are the primary causes of diabetes mellitus (DM). Nevertheless, the underlying molecular mechanisms by which β-cell growth and function are controlled are not fully understood. In this work, we show that leucettines, known to be DYRK1A kinase inhibitors, can improve glucose-stimulated insulin secretion (GSIS) in rodent β-cells and isolated islets, as well as in hiPSC-derived β-cells islets. We confirm that DYRK1A is expressed in murine insulinoma cells MIN6. In addition, we found that treatment with selected leucettines stimulates proliferation of β-cells and promotes MIN6 cell cycle progression to the G2/M phase. This effect is also confirmed by increased levels of cyclin D1, which is highly responsive to proliferative signals. Among other leucettines, leucettine L43 had a negligible impact on β-cell proliferation, but markedly impair GSIS. However, leucettine L41, in combination with LY364947, a, a potent and selective TGF-β type-I receptor, significantly promotes GSIS in various cellular diabetic models, including MIN6 and INS1E cells in 2D and 3D culture, iPSC-derived β-cell islets derived from iPSC, and isolated mouse islets, by increased insulin secretion and decreased glucagon level. Our findings confirm an important role of DYRK1A inhibitors as modulators of β-cells function and suggested a new potential target for antidiabetic therapy. Moreover, we show in detail that leucettine derivatives represent promising antidiabetic agents and are worth further evaluation, especially *in vivo*.

## Introduction

Diabetes is considered an epidemic of the 21st century by the WHO due to its worldwide prevalence and increasing mortality [1]. Its hallmark is the progressive destruction of pancreatic β-cells localized in the islets, leading to insulin insufficiency, hyperglycemia, and metabolic collapse of the body [2, 3]. The use of insulin transforms this lethal disease into a chronic disorder but does not cure it. In addition to continuous insulin supplementation, metformin is the main drug in the treatment of diabetes. However, in most patients, after long-term treatment, metformin use in monotherapy is found to be insufficient and it becomes necessary to include another drug [4]. Other available drugs do not slow the progression of diabetes and are associated with several significant side effects (weight, risk of hypoglycemia, gastrointestinal intolerance). Recognition of the mechanisms responsible for the regulation of insulin secretion and the maintenance of pancreatic β-cell function in diabetes offers hope for the development of effective therapies. The current state of knowledge indicates that a new, improved strategy to restore β-cell function is necessary for therapeutic success [5, 6].

Dual-specific tyrosine-(Y) phosphorylation regulated kinase 1A DYRK1A has been shown to negatively affect β-cell proliferation and activity. Therefore, it provides a target for designing inhibitors that will enable β-cell regeneration—leading to a reduction in insulin producing cell loss in type I diabetes (T1D) and increased insulin production in type II diabetes (T2D) [7, 8]. Last decade, high-throughput small-molecule screening (HTS) revealed that small-molecule analogues of harmine could induce β-cells proliferation [9–11]. It was also identified that the DYRK1A pathway, likely the target of harmine and the activator of nuclear factors of the activated T cell (NFAT) transcription factor family, is capable of mediate beta cell stimulation and their proliferation [9, 12, 13].

In general, DYRK1A inhibitors are divided into type I—ATP competitive inhibitors that block DYRK1A by binding to its ATP-binding domain, and types II and III, which are non-competitive inhibitors against ATP, and both act through allosteric mechanisms but differ in the way they bind to the pocket. Type II inhibitors partially bind to the target, whereas type III inhibitors are more specific than others [14]. These inhibitors induce conformational changes in DYRK1A that lead to loss of its function [15]. Studies have shown that several natural products, including harmine, epigallocatechin-3 gallate (EGCG), leucettines, and their derivatives inhibitDYRK1A kinase. On the other hand, many synthetic inhibitors with indazole, azaindole, or benzothiazole moieties have also been described with potential therapeutic activity in neurodegenerative diseases, including Alzheimer’s (AD) and Parkinson’s disease [16]. DYRK1A inhibition can also be useful for the treatment of osteoarthritis, viral infections, leukemias, and solid tumors (e.g., glioblastoma and pancreatic cancer) [17]. These considerations have motivated many investigators to seek and develop pharmacologically active DYRK / CLK inhibitors [16, 17].

To date, many DYRK1A inhibitor therapies have been reported by researchers, among which EGCG completed clinical trials in Down Syndrome in February 2010 (NCT01394796) [18, 19]. ECGC is one of the major polyphenolic components extracted from tea (especially green tea), with proven inhibitory effects on many enzymes and signaling pathways [20]. Moreover, ECGC plays an important role in many diseases, including nervous system disorders, cancer, vascular disorders, etc. [20]. As the importance of DYRK1A in numerous diseases has been increasingly recognized, work on DYRK1A inhibitors has bloomed in recent years [21, 22].

Among DYRK1A inhibitors, leucettines are extensively investigated inhibitors derived from the natural product leucettamine B produced by the marine calcareous sponge *Leucetta microraphis* [23–25]. Throughout decades, the Meijer group and Perha Pharmaceuticals & ManRos Therapeutics have characterized the structure−activity relationship (SAR) of a series of selected leucettines (>500 synthesized leucettines) differ in the substitution pattern [26, 27]. Among others, leucettine L41 has been shown to be a most promising kinase inhibitor [28]. L41 inhibits kinases of the DYRK and CLK families, and acts together with GSK3β and CK2 in an ATP competitive way. In addition, it can trigger many cellular responses such as: (i) affecting pre-mRNA splicing, (ii) protecting of HT22 hippocampal cells against cell death, (iii) inducing autophagy and inhibiting tau phosphorylation [29, 30].

Together, the results provide clues to understand the interaction of leucettines with various targets (especially CLK1, CLK4, DYRK1A, DYRK1B, and DYRK2) and suggest molecular mechanisms of action involved in the various cellular effects triggered by this type of inhibitors [31]. They also provide directions for further optimization of this family of kinase inhibitors [26]. Finally, they suggest that some leucettines favourably combine several molecular and cellular effects, supporting their development for the treatment of various clinical indications.

All of the above-mentioned issues prompted us to investigate the role of selected leucettines as known DYRK1A inhibitors as regulators of β-cell function. In the present study, we demonstrate the capability of selected leucettines to improve the glucose-stimulated insulin secretion process in rodent β-cells (MIN6 and INS1E), hiPSC-derived islets and islets isolated from BALB/c mice. These results are correlated with the inhibitory efficacy of the compounds against the selectivity of the DYRK1A kinase and the proliferation of human β-cells. Our results indicate that leucettine L41 markedly enhanced insulin production in both hiPSC-derived islets and isolated mouse islet models when compared to non-treated control groups. Moreover, this effect could be further improved by addition of LY364947 (a selective ATP-competitive inhibitor of TGF-β). Wang et al. recently reported this effect not only for leucettine L41, but also INDY and harmine and highlighted the synergy between DYRK1A and TGF-β in promoting β-cell restoration [32]. Studies show that small molecule inhibitors of DYRK1A can stimulate β-cell proliferation and improve their functionality. Thus, this strategy is promising for the development of novel methods for the treatment of diabetes, including curative therapies.

## Materials and methods

### Cell culture

In this work, two rodent β-cell lines were used. The first is the MIN6 line, produced from a transgenic mouse that expresses the large T antigen SV40 in pancreatic β-cells. MIN6 cells are characterized as susceptible to glucose-stimulated insulin secretion (GSIS response) [33, 34]. The second cell line is the INS1E rat insulinoma, a well-known and suitable model for studying insulin secretion, its regulation and pancreatic islet function [35].

MIN6 were cultured in high glucose DMEM (Pan Biotech GmbH, Germany, P04-03591) supplemented with 15% FBS (Pan Biotech GmbH, Germany, P30-19375), 55 μM β-mercaptoethanol (Pan Biotech GmbH, Germany 31350010) and 1% antibiotics (penicillin and streptomycin, Pan Biotech GmbH, Germany P06-07100). INS1E cells were grown under analogue conditions, but with 10% FBS. All of cultured were kept in incubators with 37°C and 5% CO_2_. The cells were maintained in the logarithmic growth phase throughout all studies. Every two days, the medium was replaced and cells were split and passaged with 0.25% trypsin-EDTA (Gibco, ThermoFisher Scientist, United States, 15090046).

### Cytotoxicity assay

The cytotoxicity of the selected leucettines was tested using the MTT (3-(4,5-dimethylthiazol-2-yl)-2,5-diphenyltetrazolium bromide) assay (Invitrogen, ThermoFisher Scientist, United States, M6494). After seeding cells, the compound solution was prepared in culture medium at various concentrations ranging from 0 to 100 μM (total DMSO did not exceed 5% in each case). In the second experiment, the 5 μM TGF-β inhibitor LY364947 was added in addition to the test compounds. Cells were incubated with the compounds for 24 h, after which the solution was removed, and fresh culture medium was added. After 72 h, MTT was added to each well to equal 10% of the final solution and the plates were kept in RT for 3–4 h. After this time, the medium was discarded, and the formazan crystals were dissolved in 100 μL of DMSO/methanol mixture (1:1). The absorbance measurements were performed at 565 nm using a microplate reader (Infinite M200 Reader Tecan).

### Reverse transcription and RT-PCR

Total RNA was isolated using Total RNA Mini Plus (A&A Biotechnology, Gdansk, Poland, 036–100) according to the manufacturer’s protocol. RNA (1μg) was reverse-transcribed into cDNA using oligo(dT)18 and TranScriba Kit (A&A Biotechnology, Gdansk Poland, 4000–100). qRT-PCR reactions were performed using the Sensitive RT HS-PCR Mix (A&A Biotechnology, Gdansk, Poland, 2017-2000P) and the real-time PCR thermal cycler (Bio-Rad). All TaqMan primers were from Thermo Fisher Scientific/Invitrogen: DYRK1A (Mm00432934_m1), GAPDH (cat. no. 4352932E). The relative levels of the transcripts were quantified by the 2−ΔΔCt method, using GAPDH as a reference gene.

### Glucose-stimulated insulin secretion (GSIS)

24 h before the experiment, the cell culture medium was replaced by a low glucose medium (5.5 mM). After 24 h, the medium was changed to Krebs Buffer with low glucose (2 mM). After 2 h, the medium was replaced with Krebs buffer containing 25 mM glucose for 30 min. At each step of the GSIS procedure, media and cells were collected and applied for further examination (flow cytometry, microscopic imaging, etc.).

### Staining for insulin, Ki67, glucagon, C-peptide and DYRK1A –fluorescence microscopy

Insulin, Ki67, C-peptide, glucagon, and DYRK1A were evaluated in previously selected MIN6 and INS-1E cells. First, cells were seeded on microscopic slides (1x10^5^ cells/slide) and cultured for 24 h. The cells were then treated with a 5 μM DYRK1A inhibitor for 24 h. The medium was replaced with fresh medium and after 72 h, staining of the slides was performed. Cells were fixed and then washed and permeabilized with 0.1% Triton X-100, followed by incubation with specific antibodies. Hoechst33342 (Sigma-Aldrich, United States 14533-100MG) was added at the end of the staining procedure. The cells were rinsed several times with HBSS (Gibco, ThermoFisher Scientists, United States, 14175095). Cells were imaged and visualized with a Zeiss LSM 880 fluorescence laser-scanning confocal microscope (Zeiss, Jena, Germany) and captured images were processed using ZEN software (Zeiss).

### Flow cytometry analysis

Insulin (Abcam, Germany, ab181547), Ki67 (Abcam, Germany, ab16667), C-peptide (Cell Signaling Technology, United States 4593), glucagon (ThermoFisher Scientists, United States, PA5-13442), DYRK1A (Invitrogen, ThermoFisher Scientists, United States, PA5-95499), and cyclin D1 (Abcam, Germany, ab16663) were evaluated in MIN6 and INS-1E cell lines. MIN6 and INS1E cells were seeded on round slides at a density of 10^5^ and cultured in an incubator at 37°C/5% CO_2_ for 24 h. The cells were then treated with 5 μM solutions of the appropriate leucettine or harmine for another 24 h. Cells were detached by trypsinization 48 h later, fixed, permeabilized and stained with the appropriate antibodies according to the manufacturer’s instructions, and analyzed by flow cytometry. A BD Accuri c6 flow cytometer was used for measurements and data analysis was performed using FlowJo software (FlowJo LLC based in Ashland, Oregon, Becton Dickinson).

### Cell cycle analysis

A serum starvation protocol was used to synchronize cell cultures. For this purpose, cells were seeded in culture medium containing 20% FBS and incubated overnight. Next, MIN6 cells were rinsed with PBS (with magnesium and calcium ions) and the fresh medium without FBS was added for another 24 h. After this time, the 5 μM solution of leucettine or harmine prepared in standard media (10% FBS) was added and the cells were kept in the incubator for next 24 h. Then, MIN6 cells were detached and collected using trypsin and stained with propidium iodide solution (50 μg/mL), (eBioscence, ThermoFisher Scientists, United States, 00-6990-50) for 10 min. After washing with PBS, cells were analyzed using a BD Accuri C6 flow cytometer and the obtained data were analyzed using FlowJo software.

### Western blot analysis

MIN6 cells (0.5∙10^6^/well) were cultured in 6-well microplates. After 24 h, a solution of inhibitor a concentration of 5 μM (prepared in culture medium with 0.1% DMSO). After incubation, the inhibitor was removed, and cells were washed with PBS with Ca^2+^ and Mg^2+^. After this time, cells were trypsinized and harvested. Cells were then lysed using RIPA buffer (Tris base 50 mM, NaCl 150 mM, NP40 1%, sodium deoxycholate, 0.25%, EDTA 1 mM) with the addition of proteases and phosphatases inhibitors (Sigma Aldrich, United States, 11873580001). The protein concentration in the samples was determined using the Bradford reagent and verified with NanoDrop (Thermo Scientific) measurements. In the next step, samples were prepared for gel electrophoresis (SDS-PAGE). The prepared samples with equal amounts of protein (5 μg) were resuspended in loading buffer containing (65.8 mM Tris-HCl, pH = 6.8; 26.3% (w/v) glycerol, 2.1% SDS, 0.01% bromophenol blue, 0.05 β-mercaptoethanol) and incubated in a thermoblock for about 5 min at temperature. 95°C. After denaturation, samples were applied to successive wells of polyacrylamide gel (12% gel separating gel, 6% stacking gel). Electrophoresis was carried out according to the Laemmli method (electrode buffer—running buffer: 25 mM Tris, 0.2 M glycine, 0.1% SDS) in a Protean III apparatus (Bio-Rad) at 100 V until the analyzed samples entered the separating gel, followed by 160 V for another 40 min. To prepare samples for immunoblotting, gels were transferred to nitrocellulose membranes (Bio-Rad) by electrotransfer. The gels after electrophoresis and the membranes were placed using the "sandwich" method in a transfer cassette, and then placed in an electrotransfer apparatus containing transfer buffer (transfer buffer: 25 mM Tris base, 192 mM glycine, 10% MeOH). Electrotransfer was carried out at a reduced temperature for 1.5 h, at a voltage of 100 V). After transfer, the membrane has blocked the membrane in TBS-T buffer [Tris base buffer (pH 7.4) + 0.05% Tween 20] with 2% BSA addition for 1 h (RT). After blocking, the membranes were incubated for 12 h (at temp. 2–8°C) with primary antibodies: anti-cyclin D1 (Invitrogen, ThermoFisher Scientist, United States, MA1-10324), anti-phospho(Thr286) cyclin D1 (Invitrogen, ThermoFisher Scientist, United States, PA5-104551), anti-GAPDH (Invitrogen, Invitrogen, ThermoFisher Scientist, United States, PA1-16777) in TBS-T+2% BSA. After incubation, the membranes were washed extensively in TBS-T (3x15 min on a rotor), and then incubated with a secondary antibody conjugated with horseradish peroxidase (HRP) (Invitrogen, ThermoFisher Scientist, United States, 32460). After 2 h, the membranes were washed in TBS-T and the substrate (Clarity Western ECL Substrate, Biorad). After 5 min of incubation, the membrane was visualized using the Azure 600 Gel Imaging System (Azure Biosystems). Strands were analyzed with ImageJ and the data were compiled using excel.

### Insulin quantification ELISA assay

The ultra-sensitive insulin ELISA assay (CrystalChem, United States, 90080) was performed to determine the level of total insulin content in hiPSC-derived islets and isolated islets from mice. After harmine or leucettine treatment, hiPSC-derived organoids and mouse islets were lysed using RIPA buffer with protease and phosphatase inhibitors (Sigma Aldrich, United States, 11873580001). Lysates and media obtained following the GSIS protocol were analyzed for total protein levels. Then, they were processed according to the manufacturer’s protocol for insulin level determination.

### Isolated islets

The detailed procedure was previously described by Barzowska et al. [36] and Szot et al. [37] The experiments performed were accepted by the Second Local Institutional Animal Care and Use Committee (IACUC) in Krakow (approval no 55/2022). Twelve-week-old male mice were thoroughly washed in 70% EtOH before being sacrificed by cervical vertebrae dislocation. The hemostatic clamp was inserted into both sides of the intestines. A tiny cavity was made to facilitate the infusion of collagenase IV solution (0.5 mg/mL in HBSS, Sigma Aldrich, United States, H6648). The pancreas was then injected with 3 mL of ice-cold collagenase solution via the bile duct. The pancreas was dissected and kept in 50 mL Falcon tube containing 2 mL of collagenase IV solution (ThermoFisher, United States, 17104019). Next, it was incubated for 13 min at 37°C in a water bath, followed by shaking. After this digestion step, collagenase IV was inactivated with FBS and the obtained sample was centrifuged (30 s/300 x g). The sample was then washed three times with HBSS and centrifuged under the same conditions. After this step, Percoll (1.045 g/mL), (Sigma Aldrich, United States, P4937-25ML) was used instead of HBSS. The Percoll solution began to sediment after 5 minutes. Finally, the islets that had dropped to the bottom of the tube were collected. This process was repeated three times. Freshly isolated islets were grown in RPMI-1640 (ThermoFisher, United States, 72400047) medium supplemented with 10% FBS and 1% antibiotics. The medium was replaced daily [36].

### Mouse islet staining

The presence of insulin was assessed in the isolated mouse islets. The islets were fixed for 30 min with a 3.8% paraformaldehyde solution and rinsed with PBS. Then, a 0.1% solution of Triton X-100 was added for 90 min. The islets were then rinsed two times with PBS and incubated with 1% BSA + 0.15% Triton X-100 for 60 min. After this time, a primary anti-insulin antibody (Abcam, Germany, ab181547) was applied, and islets were incubated overnight at 4°C. After the incubation, the islets were rinsed with PBS and the secondary antibody conjugated to fluorochrome (Abcam, Germany, ab150078) was added for another 3h. At the end of the staining, Hoechst33342 was added for 10 min. The islets were washed several times with HBSS. The stained mouse islets were imaged and visualized with a Zeiss LSM 880 confocal microscope using an x63 oil objective. The registered images were then analyzed using ZEN software [36].

### 3D spheroids

The spheroids were developed from the MIN6 cell line using the hanging-drop method. To create a hanging-drop, the plate lid was removed from a culture dish and 5x10^5^ MIN6 cells in 10 μL drops were placed at the bottom of the culture dish. Aggregates were removed from the cell suspension to ensure homogeneity. The size and form of high-quality spheroids are largely determined by this procedure. The cover was then inverted onto the PBS-filled bottom chamber. MIN6 spheroids were studied and cultivated on a regular basis until they formed sheets or clusters of cells. Then, they were transferred to 24-well plates that had been precoated with Geltrex matrix (ThermoFisher, United States, A1413302) and cultured with enriched growth medium until spheroids formed. A duration of up to 7 days was considered sufficient for spheroid development while using ideal growth conditions. The toxicity of one of the chemotherapeutics was then evaluated in mature spheroids [38, 39].

### Spheroids staining

The spheroids were incubated with the investigated compounds for 24 h. Then, after 72 h, they were processed for staining. The spheroids were fixed for 15 min in 3.8% solution of paraformaldehyde (PFA), washed three times with PBS, and incubated with the solution of 0.1% Triton X-100 for permeabilization (30 min). Spheroids were then washed several times with PBS (containing Mg^2+^ and Ca^2+^) and 1% BSA+0.15% Triton X-100 solution was added for 45 min. The primary antibody (Abcam, Germany, ab181547) was then added, and incubation was carried out for 12 h at 4°C. Secondary antibody (Abcam, Germany, ab150077) was added for another 3 h. At the end of the staining, Hoechst33342 was added for 10 min. The spheroids were washed several times with HBSS. Confocal images were captured with the Zeiss LSM 880 microscope (Zeiss, Germany) equipped with a x10 objective and the achieved pictures were analyzed using ZEN software.

### Differentiation of human iPSC in pancreatic cells

Human iPSC (Gibco™, ThermoFisher Scientist, United States, A18945) were differentiated into insulin-producing cells following the procedure established for the pluripotent stem cells [40]. At day 0, 8x10^5^/well hiPSC were seeded in a 12-well plate pre-coated with Geltrex and growth in Essential 8 (e8) medium with the addition of 10 μM Y-27632 (ROCK inhibitor), (Stem Cell, United States, 72304). For the differentiation to beta-cells islets, the appropriate culture media and required components were used based on the protocol of Pellegrini [40] with smart modifications, as published by Barzowska et al. [36].

### iPSCs-derived β-cell organoids staining

In order to assess the insulin production, the β-cell organoids were fixed for 15 min using paraformaldehyde at 3.8% concentration. Then, they were washed several times with PBS. To permeabilize the membranes, in the next step the 0.1% solution Triton X-100 was added to organoids for 30 min. After this time, the organoids were rinsed three times with PBS and the blocking solution containing 1% BSA and 0.15% Triton X-100 was added for next 45 min. In the next step, the primary anti-insulin antibody (Abcam, Germany, ab181547) was added to organoids and they were incubated at 4°C for 12 h. The secondary antibody was then added for another 3h (Abcam, Germany, ab150077). At the end of the staining, to stain nuclei, Hoechst33342 was added for 10 min. Organoids were washed several times with HBSS. The stained β-cell organoids were then imaged with a Zeiss LSM 880 confocal microscope (Carl Zeiss, Jena, Germany) equipped with a 40× oil objective. The obtained images were analyzed using Zeiss ZEN software.

### Statistical analysis

All results are presented as average ± SEM, and n denotes the number of experiments. All of the experiments were carried out at least three times (N = 3). Statistical significance was tested using Statistical significance was determined by one-way or two-way ANOVA with Bonferroni post hoc test using GraphPad Prism version 5.0.0 for Windows, GraphPad Software (San Diego, California USA). Statistically significant differences between groups at p<0.05, p<0.01, and p<0.001 are indicated by *, **, and ***, respectively.

## Results

### Pharmacological inhibitors of DYRK1A

**Fig 1:**
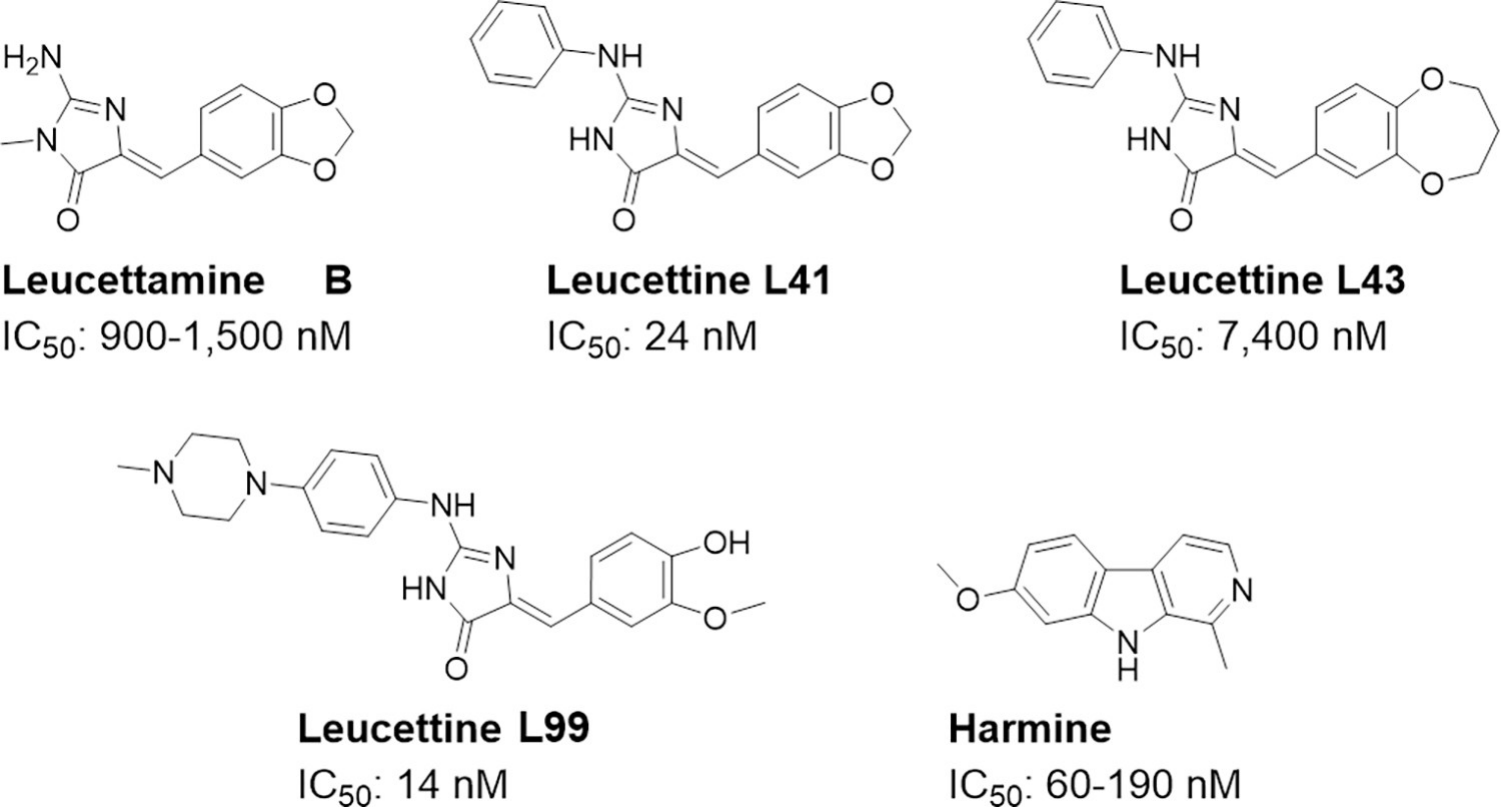
The chemical structures of the investigated leucettines. Leucettines are inspired by the natural substance, leucettamine B, produced by the marine sponge *Leucetta microraphis*. Harmine is extracted from the *Peganum harmala* plant. The IC_50_ values for DYRK1A are indicated [27].

Numerous pharmacological inhibitors against DYRK1A have been reported [15, 17, 41–46]. Among these, we selected leucettines, which represent a group of well-characterized DYRK1A inhibitors derived from the leucettamine B (marine sponge alkaloid) (Fig 1) [24, 27–29, 47–52]. Leucettine L41 and L92 are potent inhibitors of DYRK1A, while the control compound leucettine L43 is a closely related analog displaying very poor kinase inhibitory activity. We also used harmine, a frequently used DYRK1A inhibitor, despite its modest activity and several off-targets, such as monoamine oxidase [53, 54]. Most DYRK1A inhibitors have been used in the neurodegenerative context [55–57]. Our aim was to investigate some of these inhibitors for their ability to restore pancreatic β-cell functions. Our initial goal was to assess the effect of selected leucettines on β-cells in combination with the TGF-β inhibitor LY364947, as a synergistic effect with inhibition of DYRK1A had recently been described [32].

### Influence of leucettines on the viability of MIN6 and INS-1E insulinoma cells

**Fig 2:**
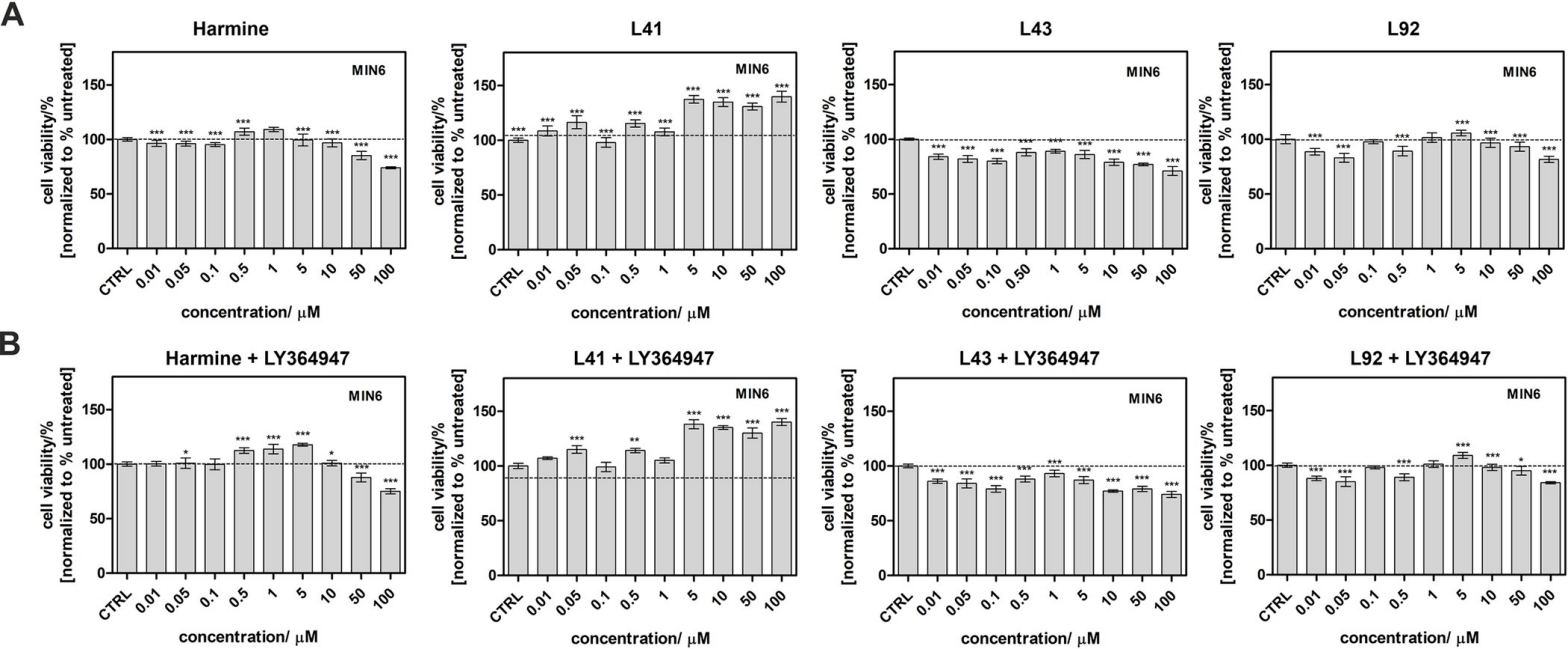
Viability of MIN6 cell lines treated with various pharmacological reagents. Cells were exposed to a range of concentrations (0 to 100 μM) of harmine or the three leucettines in the absence (**A**) or presence (**B**) of 5 μM of the TGF-β inhibitor LY364947. Cell viability, expressed as percent of the viability of vehicle-treated cells (maximum of 0.5% DMSO), was assessed with the MTT assay. Data are presented as mean ± SEM. The asterisks denote p-values < *0.05, **0.01, ***0.001 compared to the control.

We first determined the cytotoxicity of the selected compounds and their influence on β-cells viability alone and in combination with LY364947. MIN6 [58] and INS1E [35] mouse β-cell lines were treated with the vehicle, harmine as the reference compound, LY364947, leucettines, or a combination thereof. MIN6 and INS1E produce insulin and other endocrine hormones, with some showing a better response to glucose than others [33, 59–61]. MIN6 cells, a transformed β-cell line derived from a mouse insulinoma, retain GSIS and are a popular *in vitro* model for insulin secretion [62, 63]. It was also reported that MIN6 cells are not a pure beta cell line but a mixed cell line with other pancreatic endocrine hormones [64]. The second cell line used for our research—INS1E is also used in numerous studies investigating the mechanisms of glucose stimulated insulin secretion [65, 66]. As shown in Fig 2, in most cases, there was no cytotoxic effect in MIN6 and INS1E (S1 Fig) after the treatment with investigated leucettines. For leucettines L41 and L92, a significant improvement in cell viability was observed, up to 40% and 10%, respectively. Leucettine L43 and harmine showed modest cytotoxicity at high doses. As expected, the addition of LY364947 resulted in a further increase in viability, with the most pronounced effects observed for harmine at 0.5, 1, and 5 μM and for leucettine L41 (an additional 10% increase at concentrations higher than 5 μM).

### Inhibition of DYRK1A by leucettines leads to proliferation of MIN6 cells

**Fig 3:**
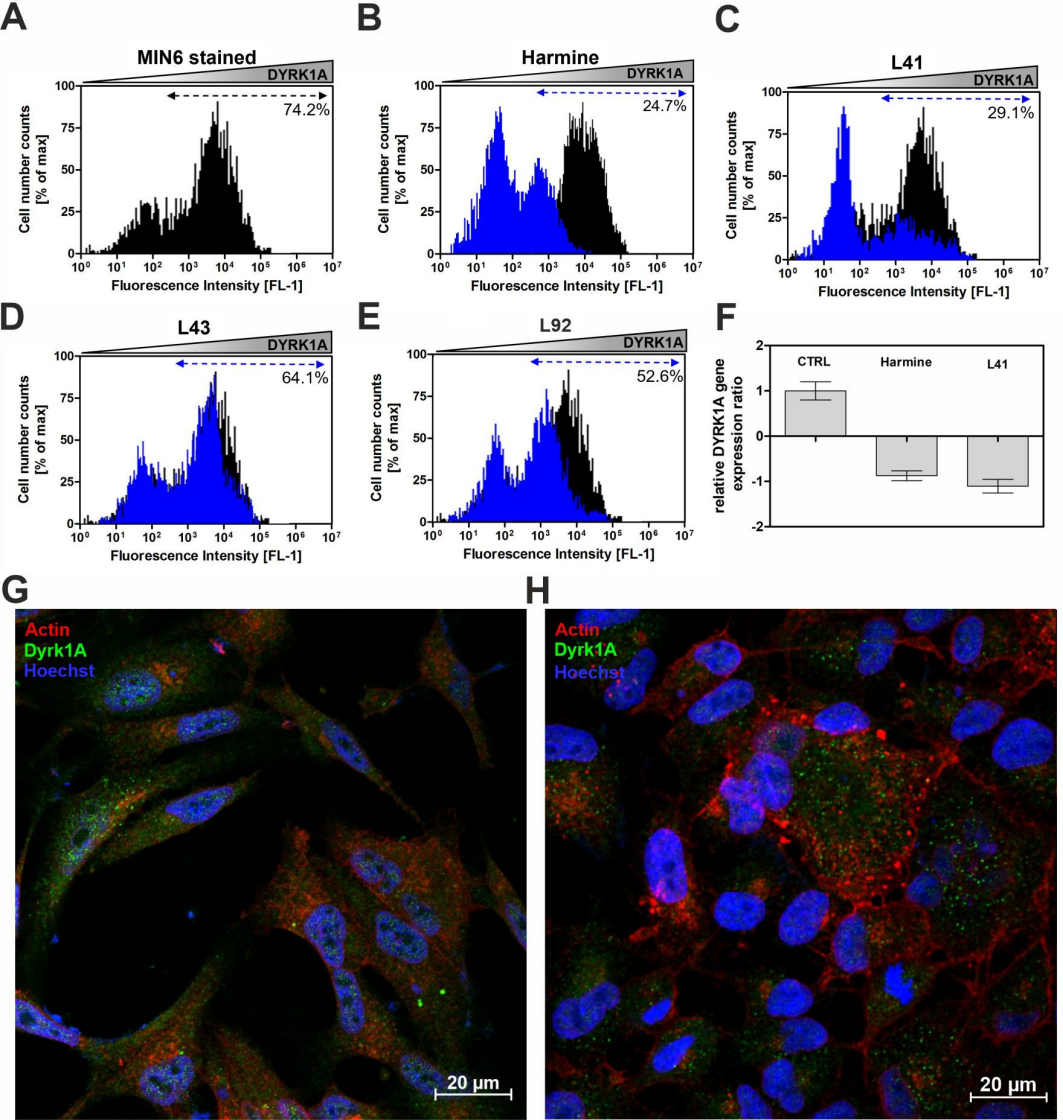
The effect of investigated leucettines on the level of DYRK1A in MIN6 cells. Flow cytometry histograms of the level of DYRK1A in MIN6 cells: **(A)** Expression level of DYRK1A in control cells. **(B-E)** DYRK1A expression levels in cells treated with 5 μM harmine, leucettine L41, leucettine L43, or leucettine L92, respectively. **(F)** RT-qPCR results were obtained for control, harmine and leucettine L41 treated cells. Confocal images for MIN6 cells stained with anti-DYRK1A antibody (green fluorescence) and cell membrane marker (red fluorescence): **(G)** cells without treatment–control cells and (**H**) cells after treatment with leucettine L41. The nuclei were counterstained with Hoechst33342 dye (blue fluorescence). Scale bars, 20 μm.

Next, we determined the expression of DYRK1A in insulinoma MIN6 cells, and the effect of selected inhibitors was investigated by flow cytometry, confocal microscopy, and qPCR analysis. Both, DYRK1A mRNA and protein were expressed in MIN6 cells. Following the addition of leucettine L41, the population of positively stained cells decreased to 29%, in contrast to the treatment with leucettine L43, where the changes were less pronounced (50–60% of stained cells vs. ~75% for untreated control cells stained for DYRK1A) (Fig 3B–3E). Similar results have been reported for harmine and other DYRK1A-specific inhibitors [36]. Real-time fluorescence PCR also showed that total DYRK1A gene expression ratios were markedly decreased upon addition of harmine and leucettine L41 compared to untreated controls (Fig 3F). As previously shown, immunofluorescence analysis confirmed the presence of DYRK1A in MIN6 cells [36]. Confocal imaging revealed that the fluorescence intensity of cells treated with leucettine L41 was weaker and was correlated with reduced expression levels of DYRK1A estimated by flow cytometry (Fig 3G and 3H).

A hallmark of diabetes is the progressive destruction of β-cells within the pancreatic islets, leading to insulin production deficiency, hyperglycemia, and metabolism breakdown. As insulin supplementation transforms this fatal disease into a chronic condition but does not cure it, restoring β-cell number and function through the stimulation of their proliferation and their insulin secretion appears as a promising alternative therapeutic strategy. To assess insulin secretion after leucettine treatment, glucose-stimulated insulin secretion (GSIS) was applied to determine glucose homeostasis and cell/islet function. GSIS is a variant of the glucose tolerance test during which the medium is sampled at key time points to measure insulin levels in the basal state and after induction of hyperglycemia by glucose. An alteration in insulin levels during a GSIS suggests an alteration in insulin secretion, which can be further analyzed by standard methods (ELISA, flow cytometry) [67].

**Fig 4:**
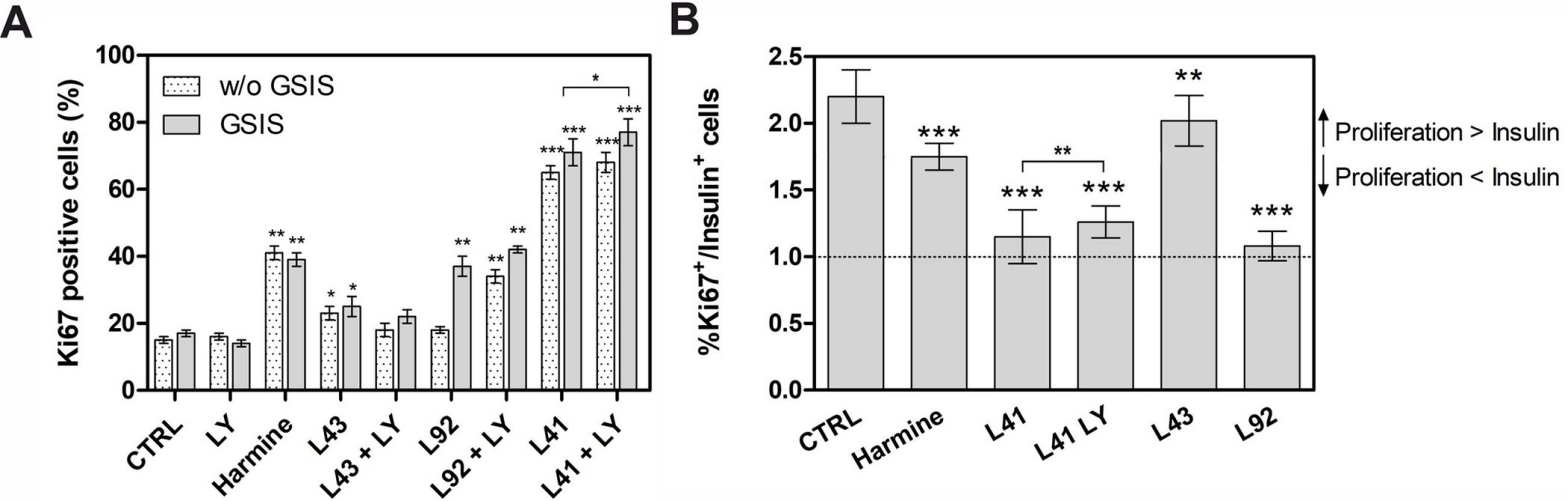
Effect of leucettines on the proliferation of MIN6 cells. **(A)** Cells were treated with 5 μM leucettines for 24 h and processed for Ki67 staining. Harmine (5 μM) was applied as a positive control. (**B**) The ratio of Ki67-positive cells to insulin-positive cells. The presented data are expressed as mean ± SEM. The asterisks denote p-values < *0.05, **0.01, ***0.001 compared to the control.

Thus, we wondered whether leucettines can induce MIN6 and INS1E proliferation by Ki67 staining, an indicator of cell proliferation (Fig 4 and S2 Fig). LY364947 did not induce any proliferation of MIN6. The poorly active DYRK1A inhibitor leucettine L43 caused very modest proliferation. In contrast, harmine and leucettine L41, alone or with LY364947, and leucettine L92 with LY364947 triggered a pronounced effect with almost 80% of Ki67 positive cells compared to 20% for untreated control cells (Fig 4A).

Next, we calculate the ’β-cell functionality index determined as the ratio of Ki67 positive cells to insulin positive cells (Fig 4B). This index can help to understand the mechanism of action of the tested DYRK1A inhibitors–leucettines and harmine (positive control). A ratio equal to 1 indicates an identical number of proliferating and insulin-secreting cells. An increase in the ratio is indicative of more proliferating cells and relatively fewer insulin-secreting cells. A decrease in the ratio suggests an effect in favour of insulin-secreting cells rather than proliferation. The results show that treatment with leucettine L92, leucettine L41, and a combination of leucettine L41 and LY364947 (ratio ca. 1.1–1.2) leads to a slightly increased population of proliferating and a higher amount of insulin-secreting cells than in case of harmine and L43. For harmine, the ratio reaches 1.7, almost two times higher than the number of proliferating cells compared to insulin-secreting cells. A similar result was obtained for leucettine L43 (almost 2.0), suggesting that this leucettine mainly stimulates the proliferation of MIN6 cells. For leucettines L92 and L41, this index is closer to 1, pointing to a lower proportion of Ki67-positive cells, compared to harmine, showing that the action of these compounds also favours insulin-secreting cells.

**Fig 5:**
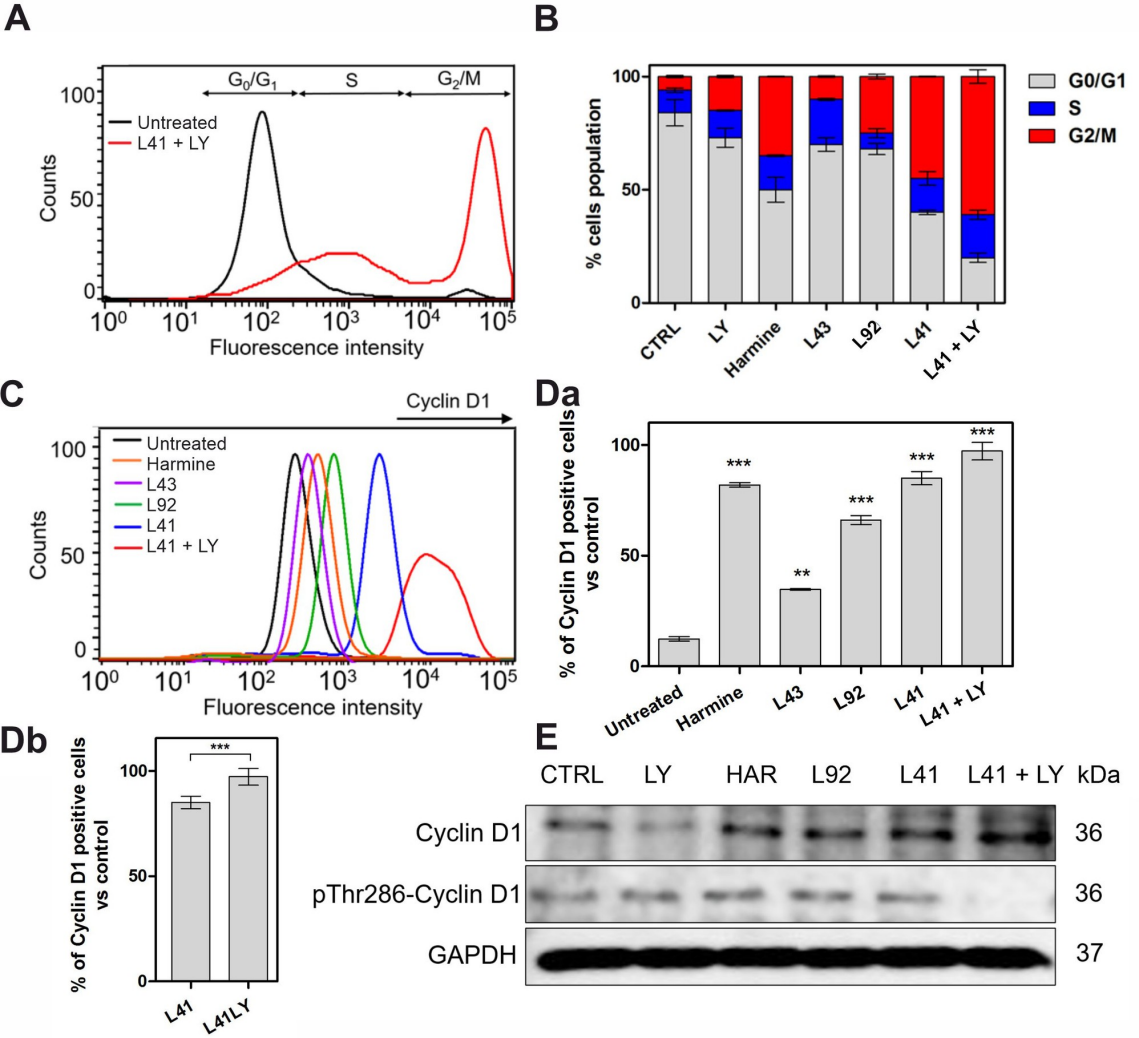
Effects of leucettines as DYRK1A inhibitors on cell cycle and cyclin D1 level. (**A**) Cell cycle of MIN6 cells analyzed by flow cytometry–representative histograms registered for vehicle-treated control cells and cells treated for 24 h with leucettine L41 and LY364947 (5 μM each). (**B**) Quantification of G0/G1, S and G2/M phase distribution based on flow cytometry analysis following the cells treatment with 5 μM leucettines. (**C**) Representative flow cytometry histograms for the level of cyclin D1 after treatment with investigated compounds. (**Da, Db**) Quantification of cyclin D1 positive cells after 24 h of treatment with each compound (based on flow cytometry analysis). **(E)** Western blot analysis of the level of cyclin D1 and pThr286-cyclin D1 in MIN6 cells after treatment with selected compounds. GAPDH was used as a reference protein. Data are expressed as mean ± SEM. The asterisks denote p-values < *0.05, **0.01, ***0.001 compared to the control.

DYRK1A has been reported to be able to inhibit the expression (or promote degradation) of members of the cyclin D family *in vitro* and *in vivo* [63]. DYRK1A phosphorylates cyclin D1 on Threonine 286 during the G1 phase, thus promoting its proteasomal degradation and subsequent cell cycle arrest in developing cells [68, 69]. DYRK1A can therefore alter cell cycle progression and further proliferation. These results support the hypothesis that DYRK1A affects β-cell function and that DYRK1A expression may contribute to diabetes development. Therefore, we examined the effects of leucettines on the cell cycle of MIN6 cells (Fig 5). In untreated control cells, the expression of DYRK1A arrests cells in the G1 phase. However, treatment with harmine and leucettines L41 and L92 altered the proportion of cells in the G0 phase and revealed a subpopulation of MIN6 cells in the S and G2/M phases (Fig 5A and 5B). The most significant effect was observed for leucettine L41, with cells in phase G2/M reaching almost 45%. The addition of the TGF-β inhibitor caused a further increase in G2/M phase cells (up to 60%).

To complete the cell cycle analysis, we stained MIN6 cells to detect cyclin D1 expression levels following drug treatments. Fig 5C and 5D show increased levels of cyclin D1 positive cells after incubation with DYRK1A inhibitors. The strongest effect was observed with leucettine L41 and leucettine L41 + LY364947. The expression of DYRK1A arrests proliferating cells in the G1 phase within approximately 24 h for neural cells (as previously reported) [69]. DYRK1A inhibition leads to stabilization of cyclin D1 in MIN6 cells, thus favoring the progression of the cell cycle into the S and G2/M phases. Furthermore, DYRK1A phosphorylates Cyclin D1 in threonine 286 (T286) to promote its degradation and subsequent cell cycle arrest in developing cells. Therefore, inhibition of DYRK1A may avoid this process and allow cells to proliferate. This effect was confirmed by Western blot analysis, which revealed that specifically treatment with leucettine L41+LY364947 decreases the level of Thr286-phosphorylated cyclin D1 (Fig 5E and S3 Fig).

### DYRK1A inhibition by leucettines leads to insulin production by MIN6 and INS-1E insulinoma cells

**Fig 6:**
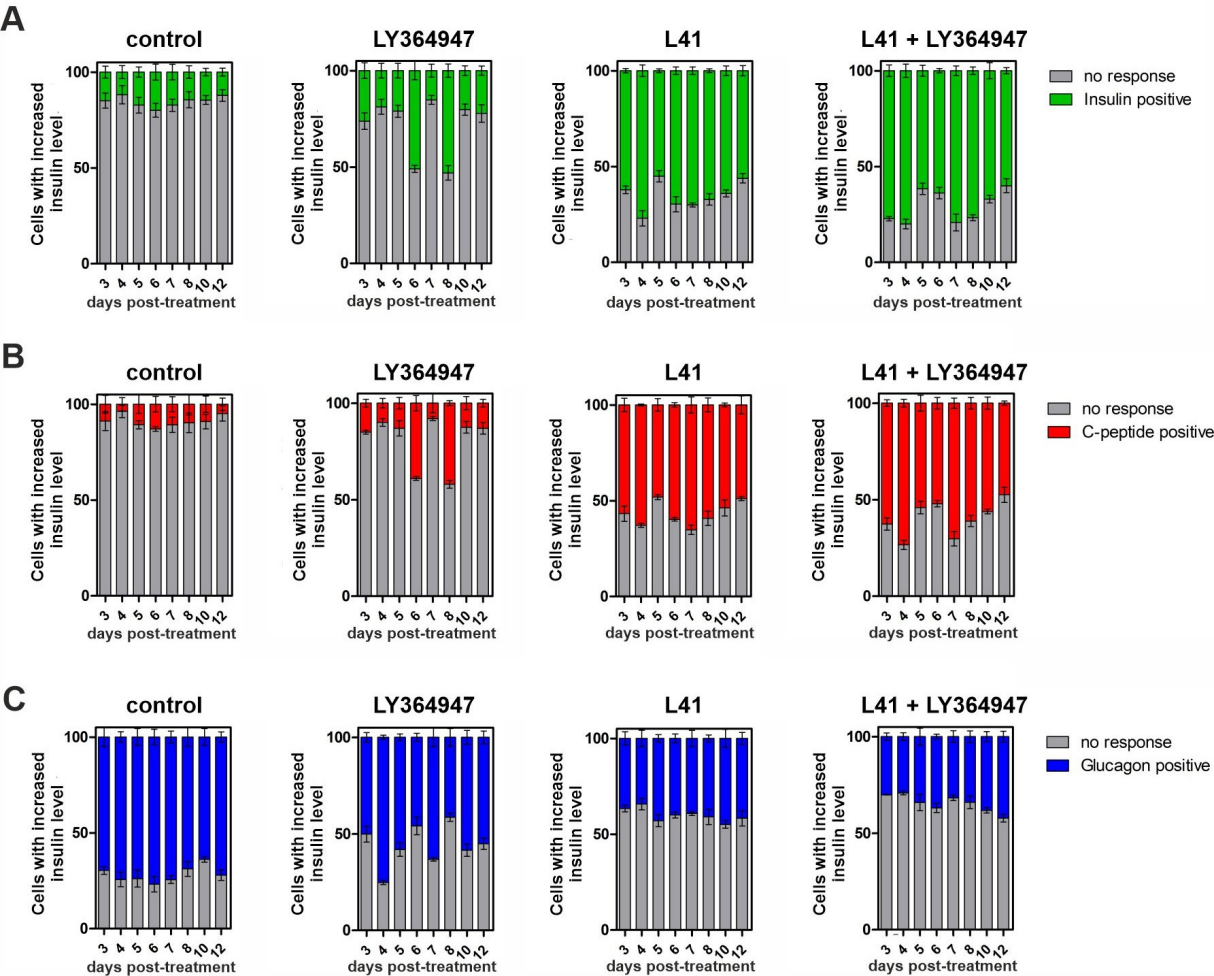
The influence of leucettines as DYRK1A inhibitors on insulin, C-peptide, and glucagon production by MIN6 cells. Flow cytometry analysis of (**A**) insulin, **(B)** C-peptide, and (**C**) glucagon levels in MIN6 cells. Cells were treated with a 5 μM inhibitor for 24 h and 72 h later, cells were stained with appropriate antibodies.

To assess whether leucettines affect insulin secretion function, we measured glucose-stimulated insulin secretion (GSIS) in MIN6 and IN S4 and S5 Figs) cells treated for 12 days with each leucettine (L43, L92, and L41) and their combination with LY364947. Cells were treated with the investigated compounds (5 μM) for 24 h. The GSIS assay was performed for the following 12 days and cells were analyzed by flow cytometry in terms of insulin, C-peptide and glucagon levels (Fig 6).

In control cells treated with vehicle, the insulin level was stable for 12 days and ~20% of cells treated with GSIS responded positively. A relatively low level of C-peptide was observed. On the contrary, these control cells expressed a high level of glucagon with ~70% positively stained, suggesting a possible impaired glucose tolerance in untreated insulinoma cells. It is noteworthy that, the pathophysiology of T2D is characterized by altered glucagon secretion, resulting in higher glucagon concentrations in both fasting and post-meal states [70]. In cells treated with LY364947, an approximately 3-fold increase in insulin secretion was observed on days 6 and 8 after the addition of LY364947. The C-peptide-positive cells changed in a similar manner. Furthermore, on these two days indicated, the glucagon level decreased by 20% compared to the control, consistent with the general fact that glucagon antagonises insulin action. Interestingly, leucettine L41 caused the most pronounced changes in hormone secretion. The insulin-positive cells population increased to 70% and reached a maximum value 4 days after treatment (vs. 15% for control cells and 25% for LY364947-treated cells) and the high insulin level (more than 50%) persists on all subsequent days of the experiment. The number of C-peptide-positive cells was correlated with that of insulin. On the contrary, the glucagon-positive cell population was markedly reduced, its level never reached over 50% on any of the days analysed after treatment. Furthermore, these effects were more prominent for cells treated with leucettine L41 and LY364947. The peak in insulin production occurred as early as day 3 after treatment with the combination of the DYRK1A and TGF-β inhibitors. As expected, the population of C-peptide-positive cells corresponded well with the population of insulin-positive cells, whereas the number of glucagon-positive cells decreased.

**Fig 7:**
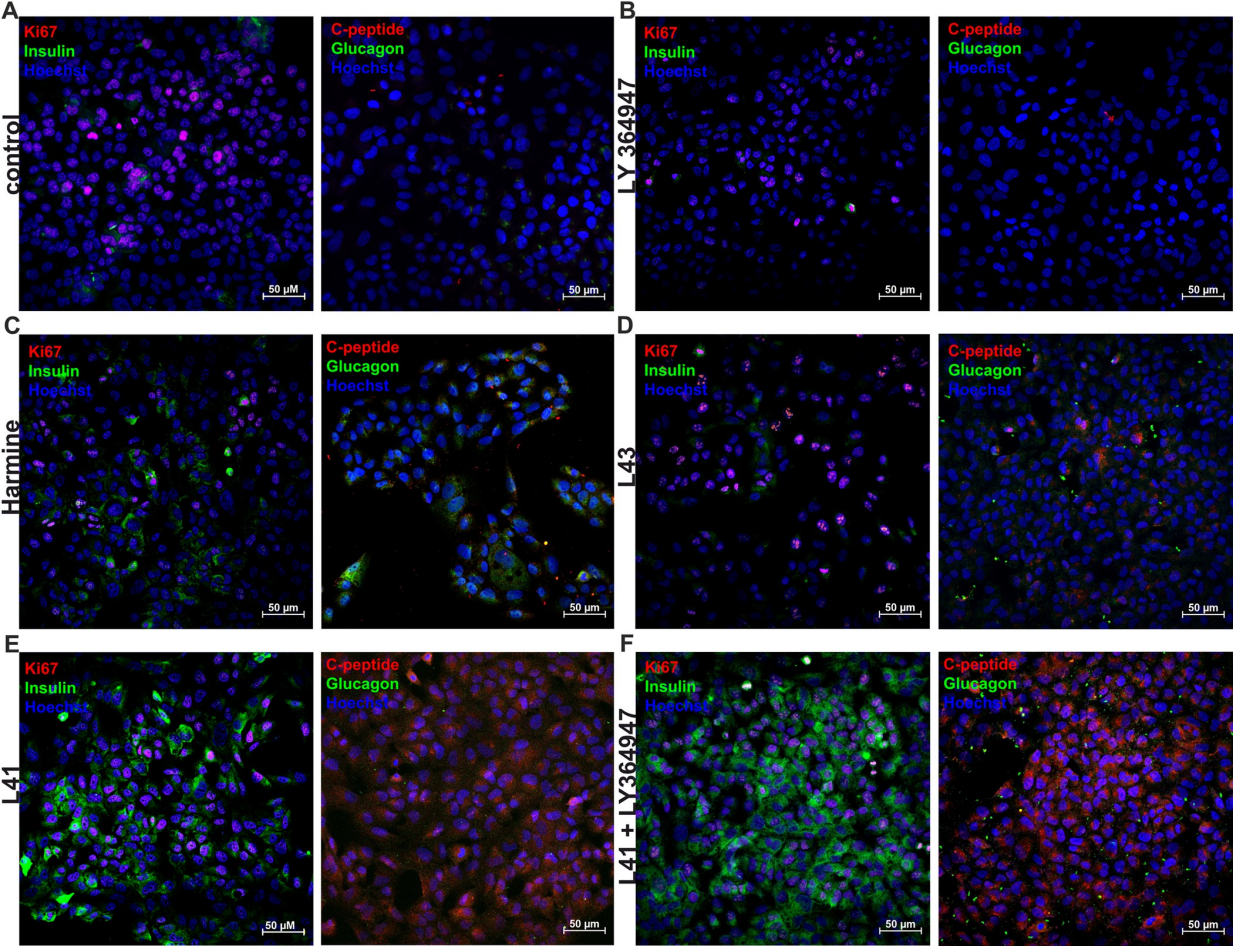
Confocal imaging of MIN6 in the context of cell proliferation, as well as insulin, C-peptide, and glucagon levels after treatment with investigated leucettines. The proliferation rate examined by Ki67, insulin (INS), C-peptide and glucagon levels for (A) vehicle-treated, control cells, and cells treated for 24 h with: (B) 5 μM LY364947, (C) 5 μM harmine, (D) 5 μM leucettine L43, (E) 5 μM leucettine L41, (F) 5 μM leucettine L41, and 5 μM LY364947. Scale bar—50 μm.

Confocal microscopy imaging further confirmed the relationship between insulin production, C-peptide, and glucagon (Fig 7 and S6 Fig). Control cells treated with vehicle, and LY364947 showed low levels of hormones (Fig 7A and 7B). These quietly low signals were also observed for cells treated with leucettine L43 (Fig 7D). Treatment with harmine and leucettine L41 resulted in relatively high insulin and C-peptide signals with moderate glucagon fluorescence (Fig 7C and 7E). The combination of leucettine L41 and LY364947 triggered a superior response in insulin and C-peptide production and in decreased glucagon levels (Fig 7F).

### Treatment of MIN6 3D spheroids with leucettines leads to insulin production

**Fig 8:**
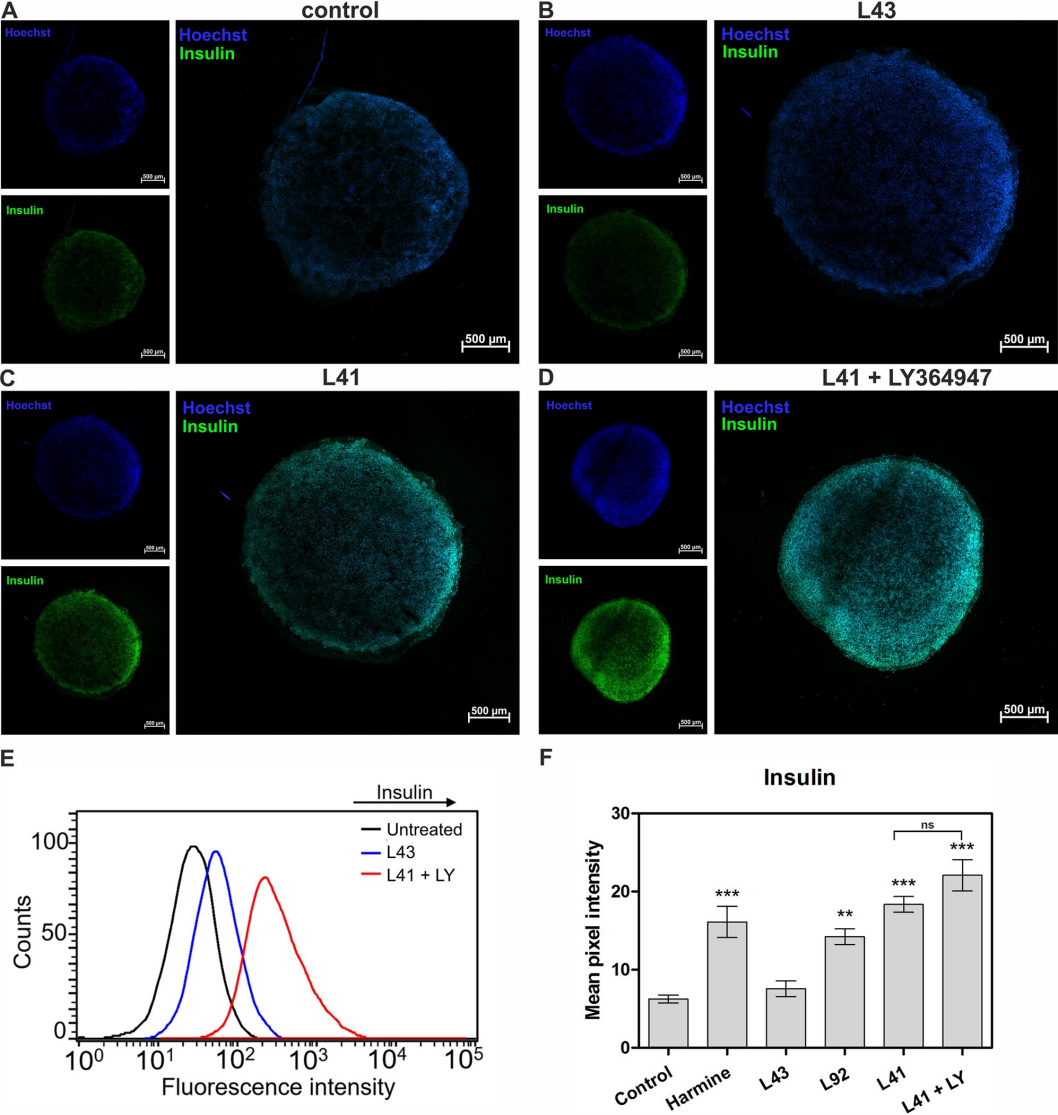
The influence of selected leucettines on insulin production by 3D MIN6 spheroids. Confocal imaging of insulin production in spheroids: representative images of insulin secretion level (marked with green fluorescence) for (**A**) vehicle-treated control spheroids and after treatment with (**B**) 5 μM leucettine L43 (negative control), (**C**) leucettine L41, (**D**) leucettine L41 and LY364749. Hoechst-33342 was used to stain nuclei (blue fluorescence). (**E**) Flow cytometry analysis of insulin levels after treatment with leucettine L43 or leucettine L41 and LY364749, compared to vehicle-treated cells. (**F**) Insulin expression level as mean pixel intensity based on image analysis for all investigated compounds. Scale bars, 500 μm. Data are expressed as mean ± SEM. The asterisks denote p-values < *0.05, **0.01, ***0.001 compared to the control.

Established β-cell lines, including MIN6 and INS1E, represent a good model to study β-cell physiology using conventional two-dimensional (2D) culture procedures. Although these cell lines are widely used, it should be noted that the stability of immortalised β-cell cultures gradually decreases over time due to phenotypic alterations induced by continuous growth controlled only by passages and long-term culture. Therefore, 3D models offer an alternative to standard 2D in vitro culture that allows not only to mimic the microenvironment *in vivo*, but also long-term dynamic cultures, with proper cell-to-cell interactions and more tissue-specific morphology [59, 71, 72]. Therefore, in this work, in addition to testing MIN6 cells growing in 2D culture, we also used a 3D spheroid model. Spheroids were prepared using the hanging-drop method, and β-cell function was assessed by determining insulin levels in response to glucose stimulation. After optimal growth, the spheroids were stained and visualized by confocal microscopy imaging. As shown in Fig 8, 3D spheroids of MIN6 cells also indicated glucose sensing and insulin secretion ability, similarly to 2D monolayer cultures. The fluorescence intensity detected for insulin was found to increase in the following order according to the different treatments: control spheroids = leucettine L43 < leucettine L41 < leucettine L41 + LY364947 (Fig 8A–8D). Flow cytometry analysis of spheroids revealed a large shift for insulin staining following leucettine L41 + LY364947 treatment compared to vehicle-treated control cells (Fig 8E). Image analysis of insulin-positive cells staining confirmed the increased insulin fluorescence signal in spheroids treated with leucettine L41 and leucettine L41 + LY364947 (Fig 8F).

### Treatment with leucettines of hiPSC-derived pancreatic islet-like organoids leads to improved insulin production

Recent advances in stem cell technology have demonstrated the considerable potential of induced pluripotent stem cells (iPSC) and β-cells organoids of cells as next-generation tools for *in vitro* investigation of diabetes pathology and drug evaluation. Pancreatic islet-like organoids are now generated from reprogrammed mononuclear blood cells into iPSCs, which are then differentiated into islet-like mini-organs [73–75]. The protocols for generating these organoids are still being developed but work in this area is progressing rapidly.

**Fig 9:**
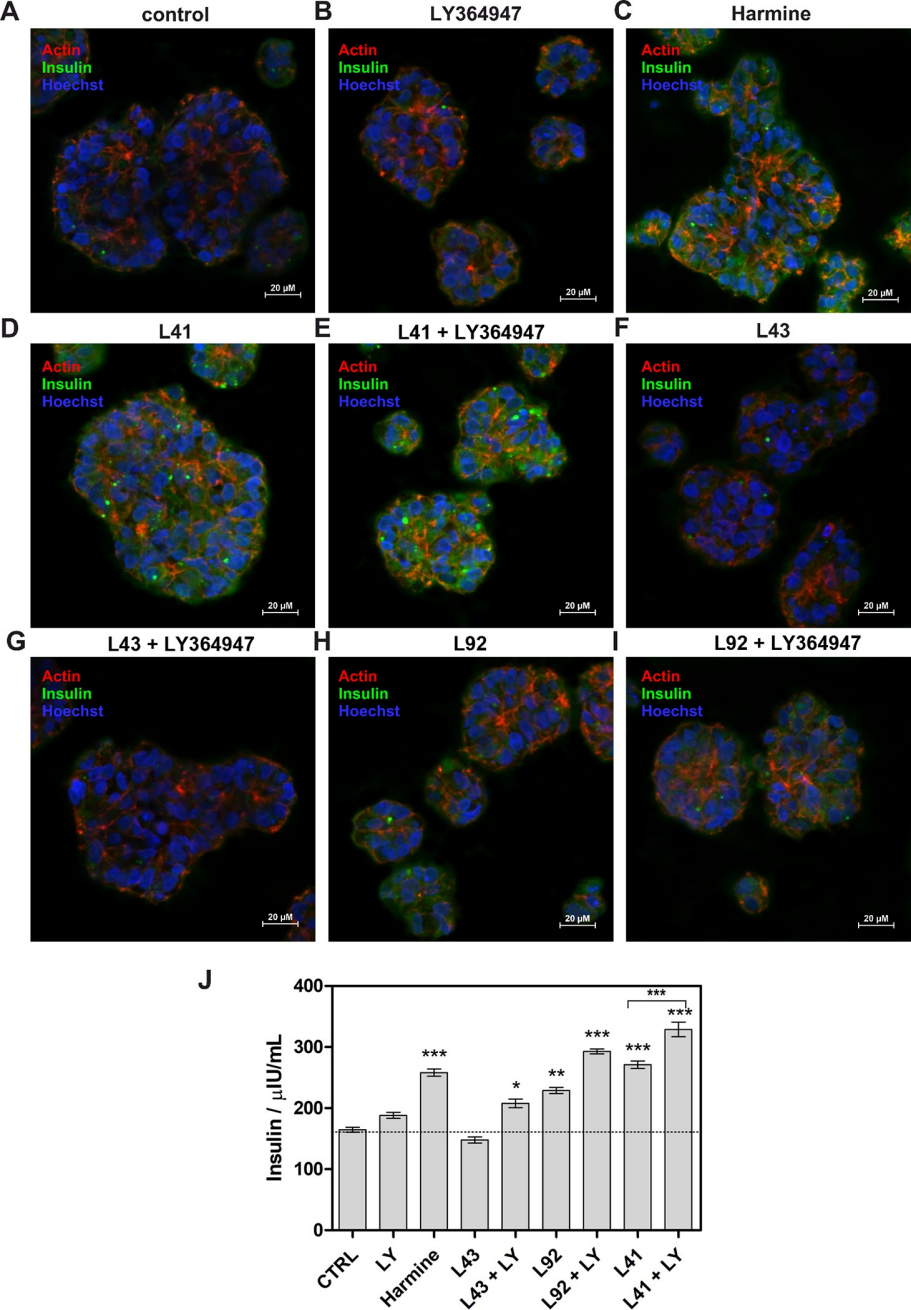
Development of hiPSC-derived β-cells organoids and the effect of leucettines on the insulin production. (**A-I**) Representative images of β-cells organoids organoids stained for insulin and nuclei (Hoechst 33342). Organoids were treated with 5 μM leucettine L41 (**A**) or 5 μM leucettine L41 + 5 μM LY364947. (**J**) Insulin level analysis after treatment with various compounds (5 μM inhibitor and 5μM LY364947) and the GSIS procedure. Scale bars, 50 μm. Data are expressed as mean ± SEM. The asterisks denote p-values < *0.05, **0.01, ***0.001 compared to the control.

The differentiation of human iPSC to beta-cell islets was performed using the Pellegrini protocol with smart modifications. The expression of characteristic biomarkers for each stage of differentiation (e.g., SOX17 (at day 5) and PDX-1 (at day 7) as well as pancreatic hormones (insulin, C-peptide, glucagon at day 14) was used to validate the progress of differentiation and maturity of organoids [36]. Mature beta cell-organoids with spherical shape and size >50 μM were treated with compounds for 24 h, followed by GSIS (Fig 9A–9I). Then, they were subjected to microscopic analysis and ELISA to quantify secreted insulin. The results clearly indicate that the organoids obtained contain functional pancreatic cells capable of producing insulin and providing a strong GSIS response (Fig 9A–9I). Furthermore, a marked increase in insulin level was observed in organoids treated with harmine, leucettine L41, leucettine L92, or leucettines combined with LY364749 (insulin content > 200 μUI/mL) compared to control cells treated with vehicle, and leucettine L43 (insulin content ~150 μUI/mL). The addition of the TGF-β inhibitor favored increased insulin secretion in all cases (Fig 9J).

### The effect of leucettines on isolated mouse islet’s function

**Fig 10:**
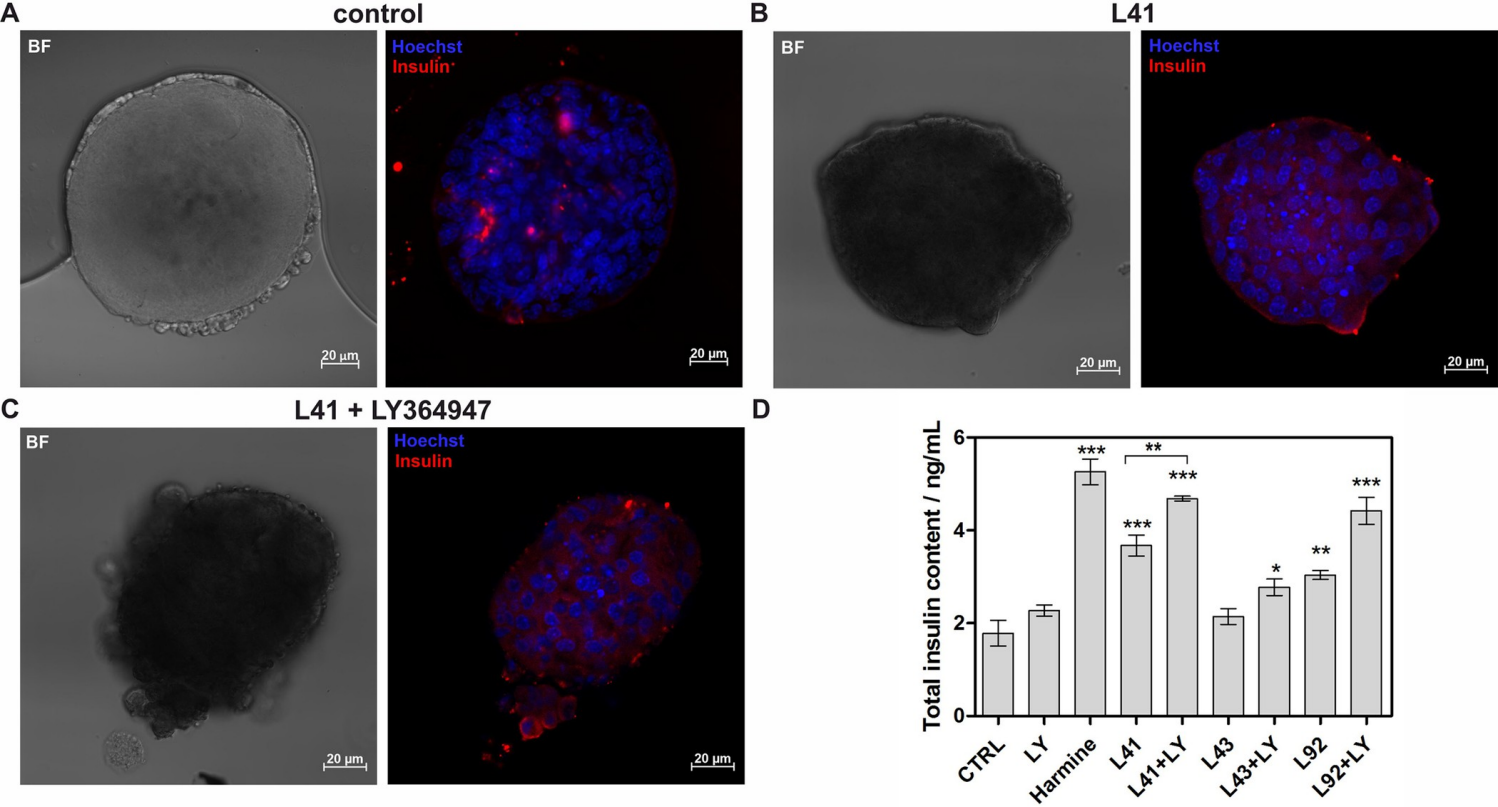
Effects of DYRK1A inhibitors on isolated mouse islets. Representative Hoechst 33342 DNA staining (blue) and insulin immunostaining (red) of isolated islets treated with vehicle (**A**), leucettine L41 (**B**), and leucettine L41 + LY364947 (**C**). Scale bars, 20 μm. (**D**) Insulin quantification. The islets were incubated with the selected inhibitors (alone or with LY364947) and GSIS was performed. Insulin levels were assayed as described in the Experimental Procedures section. The total insulin content was presented as a fold increase compared to the untreated control. Data are expressed as mean ± SEM. The asterisks denote p-values < *0.05, **0.01, ***0.001, compared to the control.

Isolated mouse islets are widely used in many studies regarding diabetes [76]. An adequate quantity of good quality islets is a prerequisite for reliable studies. Here, we used a previously optimized protocol for islet isolation from the mouse pancreas [36]. In general, there are three main steps in the islet isolation procedure: *in situ* pancreatic perfusion with collagenase, pancreatic digestion, and islet purification. Using this protocol, it was possible to obtain an adequate number of high-quality islets to analyze the effects of our compounds. Fluorescence microscopy images of islets treated with leucettine L41 improved insulin secretion in isolated mouse islets (Fig 10A–10C). Furthermore, based on the ELISA results it can be observed that harmine treatment increased the glucose-induced insulin release up to 5.26 ng/mL compared to 1.79 ng/mL for untreated control (Fig 10). Leucettine L43 indicated a small effect on glucose-induced insulin secretion at 2.14 ng/mL. The most enhanced effect was observed for leucettine L92 (3.04 ng/mL) and leucettine L41 (3.67 ng/mL) (Fig 10). After incubation with the combination of leucettines and LY364947, insulin secretion was more pronounced than for the compound alone (2.78 ng/mL for leucettine L43+LY364947; 4.42 ng/mL and 4.68 for leucettine L92 and leucettine L41 with LY364947, respectively). Thus, the combination of LY364947 with leucettine L41 again led to the strongest effect, with more than two times higher insulin secretion than control vehicle-treated islets.

In conclusion, the present study demonstrates that the combination of leucettine L41 with LY364947 acutely stimulates insulin secretion from isolated pancreatic islets in a glucose-dependent manner.

## Discussion

Attenuated insulin release from β-cells represents one of the major contributors to diabetes. At the early phase of the disease, β-cell proliferation and function are enhanced to address the augmented insulin requirement. However, the intrinsic mechanism underlying the reduction in the number of β-cells or the impairment of insulin secretion in diabetes remains largely unclear. In β-cells, insulin exocytosis is partially regulated by specific kinases, which by altering protein phosphorylation, modify the assembly of proteins associated with cell proliferation and hormone secretion (e.g., insulin, C-peptide, glucagon). DYRK1A has been shown to negatively affect β-cell proliferation and activity. Therefore, it provides a target for the design of inhibitors that might enable β-cell regeneration, thus contributing to reducing insulin-producing cell loss in type I diabetes and increasing their efficiency in type II diabetes.

Growing evidence indicates that inhibition of DYRK1A is critical for β cell restoration and confirm that the DYRK1A signaling pathway is deregulated in diabetes [8, 12, 57, 77].

Small-molecule inhibitors such as harmine, INDY, GNF4877, 5-iodotubericidin (5-IT) or CC-401 induce human beta cells to proliferate, generating a labelling index of 1.5%–3% [32].

In our previous study, we identified that the set of synthetic DYRK1A inhibitors are able to improve the functionality of beta cells in various in vitro models [36]. In these studies, we used three leucettines (L41, L92 and L43) and a reference inhibitor Harmine, which was the first identified small molecule inhibitor of DYRK1A and remains the gold standard in β-cell proliferation. Harmine, a naturally occurring compound orally bioavailable, is the best studied and a highly potent DYRK1A-inhibitor [78], and has been found to induce human β-cell-proliferation up to 3% [32, 79].

Wang et al., for the first time, explored the effects of TGFβSF pharmacologic inhibitors of TGFSF on human beta cell proliferation in a large number of human cadaveric islet preparations. They used a set of DYRK1A inhibitors including Harmine, INDY and declared that they are capable of activating human beta cell proliferation [32].

However, it was revealed that inhibition of DYRK1A and transforming growth factor beta superfamily (TGFβSF)/SMAD signaling generates further notable synergistic increases in human beta cell proliferation (average labelling index, 5% - 8% and as high as 15%–18%), and increases in both the number of mouse and human beta cells [32].

The most studied combination includes harmine with the TGFβ inhibitor or GLP-1 synergistically induced human beta cell replication in human pancreatic islets from cadaveric donors, both ex vivo and transplanted into euglycemic and immunodeficient diabetic mice. The combination of a DYRK1A inhibitor, harmine, together with exendin-4, increases human β cell mass in immunodeficient mice transplanted with human islets by 700% over 3 months of treatment, while also reversing diabetes [80].

However, harmine also acts as monoamine oxidase (MAO) inhibitor [81]. Therefore, it is a challenge to find the DYRK1A inhibitor that is selective towards beta cells and does not induce side effects. In this study, we used three leucettine derivatives as DYRK1A inhibitors to study the roles of this kinase in regulating β-cell function. We found that inhibition of DYRK1A promotes β-cells proliferation, and GSIS indicates that in β-cells, DYRK1A is necessary for normal β-cell function by directly promoting insulin secretion.

We have demonstrated that natural derivatives—leucettines can promote insulin secretion by insulinoma cells, hiPSC-derived beta islets, and isolated mouse pancreatic islets cultured *in vitro*. In MIN6 and INS1E cells, treatment with leucettine diminishes the impairment of GSIS. Furthermore, the addition of LY364947 to leucettine L41 significantly increases insulin secretion. Similar promoting effects were observed in be β -cell islets derived from hiPSC and mouse islets. This combination of drugs increases the rate of proliferation and glucose response through research, suggesting effects on signaling pathways that promote β-cell replication, insulin biosynthesis, and secretion.

In our recent articles, we select one of the best synthetic DYRK1A inhibitors (named as AC27) and evaluated its potential for beta cells (Table 1) [22, 36]. Therefore, together with our previous experience, it can be concluded that: (1) as AC27 and harmine, L41 is non-toxic in a wide range of concentration, (2) both inhibitors lead to ca. 75% of Ki67 positive MIN6 cells after treatment (better effect than for harmine), (3) both increase insulin and. The C-peptide content in MIN6 cells with a lower glucagon level, (4) AC27 and Harmine indicates the Ki + / Ins + ratio. at 1.6–1.7. For L41 this value is close to 1.2. Thus, it can be suggested that for L41 insulin stimulation is more preferred mechanisms than proliferation and for AC27 and harmine—for those proliferation is more preferred than insulin secretion; (4) in human beta cell organoids, L41 induce more insulin than harmine after gsis, when for AC27 this effect is slightly lower. Moreover, from biochemical point of view, the IC50 value for L41 is 24 nM vs 532 nM for AC27 and 60–190 nM for harmine, respectively. Similarly as for L41, AC27 prevented tau phosphorylation, demonstrating an on-target effect in vitro, compared to harmine, which indicates many off-target effects.

**Table 1:** The summary of selected properties and biological effects on beta cells determined for leucettine L41, harmine (natural, best-characterized DYRK1A inhibitor) and AC27 (synthetic, specific DYRK1A inhibitor).

		Harmine	AC27	L41
**Biochemistry**	**IC50**	30–80	532	24
	**Specificity**	No	yes	no
	**off-targets**	Yes	no	yes
**Cell lines**	**Toxicity**	No	no	no
	**Ki67** ^**+**^	**↑↑**	**↑**	**↑↑**
	**Ki67** ^**+**^ **/insulin** ^**+**^	**↑**	**↑**	**↑**
	**insulin** ^**+**^	**↑**	**↑↑**	**↑↑**
	**glucagon** ^**+**^	**↑↑**	**↑**	**↑**
	**C-peptide** ^**+**^	**↑**	**↑↑**	**↑↑**
**hiPSC**	**insulin secretion (ELISA)**	**↑↑**	**↑**	**↑**
**isolated islets**	**insulin secretion (ELISA)**	**↑**	**↑↑**	**↑**

Thus, it can be concluded that L41 as a natural product indicates similar properties to synthetic DYRK1A inhibitors. Furthermore, it causes increased insulin secretion in beta cells *in vitro* (better than harmine, the golden standard) and can potentially be used as an antidiabetic agent.

## Supporting information

S1 Fig Relative viability of the INS1E cell lines treated with investigated DYRK1A inhibitors in an MTT assay. (TIF)

S2 Fig Analysis of the influence of DYRK1A inhibitors on proliferation of INS1E cells. (TIF)

S3 Fig Protein level calculated in ImageJ from Western blot. (TIF)

S4 Fig The influence of DYRK1A inhibitors on MIN6 function: Flow cytometry analysis of insulin, C-peptide and glucagon expression in MIN6 cells. (TIF)

S5 Fig The influence of DYRK1A inhibitors on INS1E function: Flow cytometry analysis of insulin, C-peptide and glucagon expression in INS1E cells. (TIF)

S6 Fig Representative confocal images. Proliferation rate (Ki67), insulin (INS) secretion and expression levels of C-peptide and glucagon.
(TIF)

S1 Graphical abstract (TIF)

## Abbreviations

AD: Alzheimer’s disease
AMKL: acute megakaryoblastic leukemia
BBB: blood brain barrier
CDKs: cyclin-dependent kinases
CK2: casein kinase 2
CLKs: cdc2-like kinases
CB1: cannabinoid receptor 1
DMSO: dimethylsulfoxide
DS: Down syndrome
DTT: dithiothreitol
DYRKs: dual specificity, tyrosine phosphorylation regulated kinases
FBS: fetal bovine serum
GPCRs: G-protein coupled receptors
GSIS: glucose-stimulated insulin secretion
GSK3: glycogen synthase kinase-3
GST: glutathione-S-transferase
MRD7: Mental Retardation Disease 7
MTS: 3-(4,5-dimethylthiazol-2-yl)-5-(3-carboxymethoxyphenyl)-2-(4-sulfophenyl)-2*H-*tetrazolium
PBS: phosphate-buffered saline
PD: Parkinson’s disease
SAR: structure activity relationship
SRp: serine/arginine–rich proteins
T2D: type II diabetes
