# Supplementary figures and images for: DYRK1A inhibitors leucettines and TGF-β inhibitor additively stimulate insulin production in beta cells, organoids, and isolated mouse islets
Source: PLoS One. 2023 May 17;18(5):e0285208. doi: 10.1371/journal.pone.0285208 (PMC10191338; doi:10.1371/journal.pone.0285208)

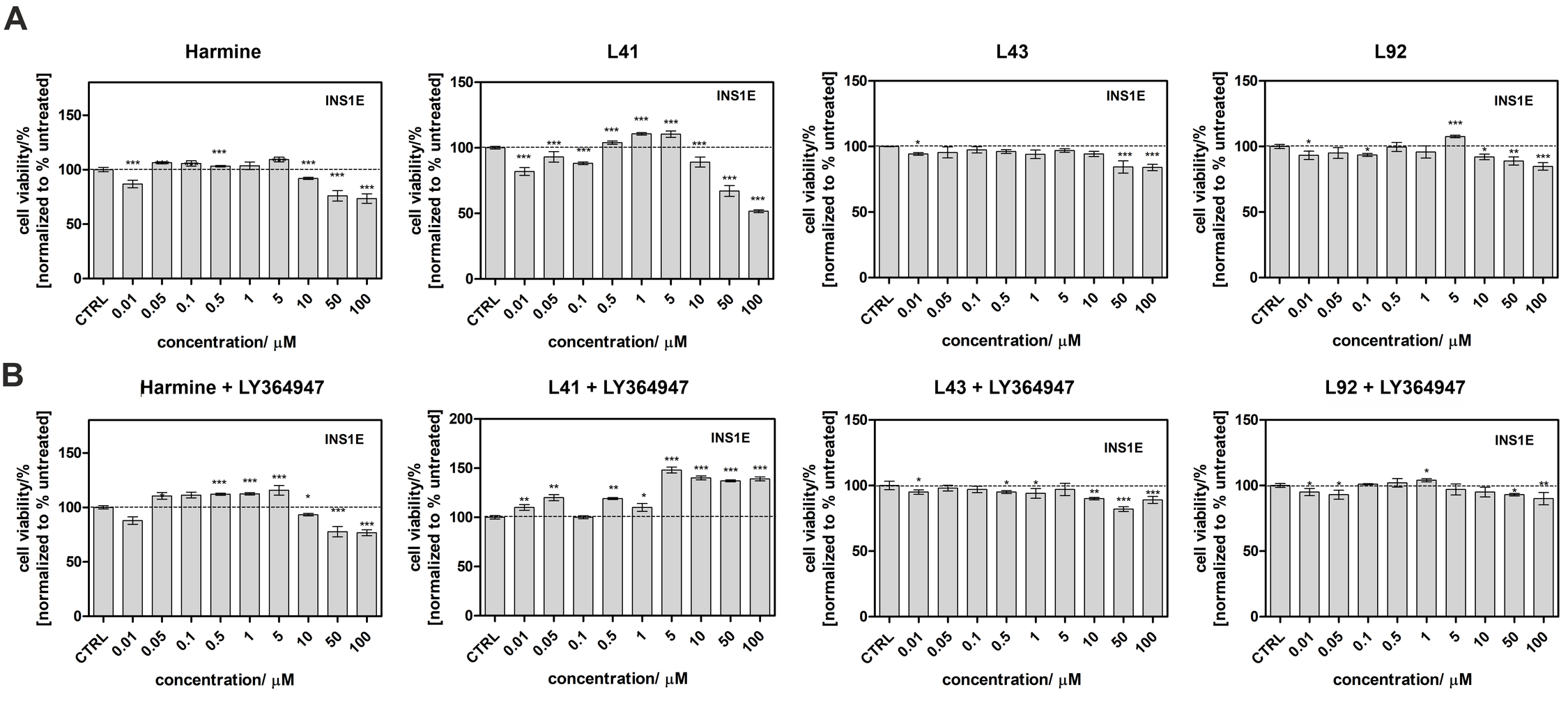

Supplement: S1 Fig — (TIF) [file pone.0285208.s001.tif]

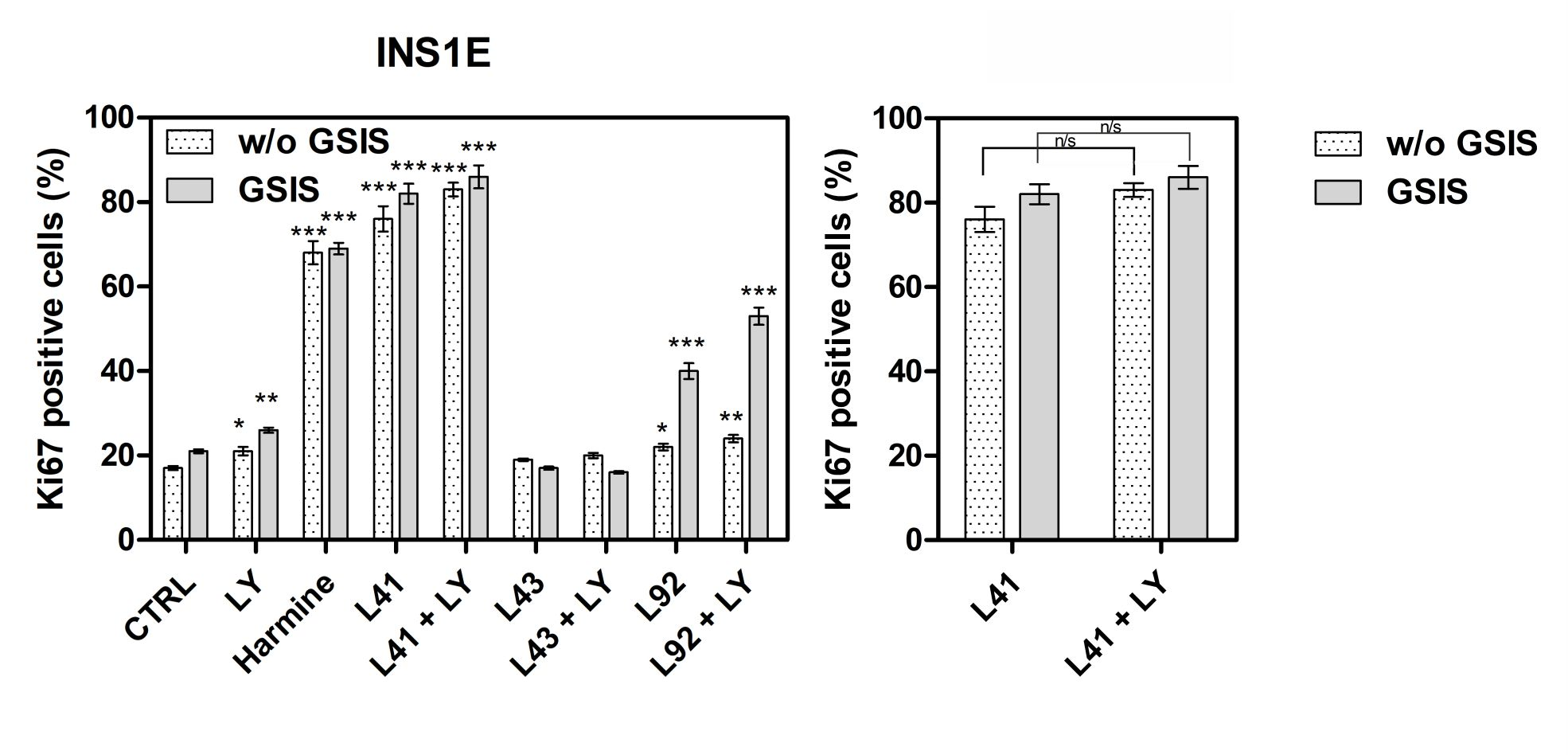

Supplement: S2 Fig — (TIF) [file pone.0285208.s002.tif]

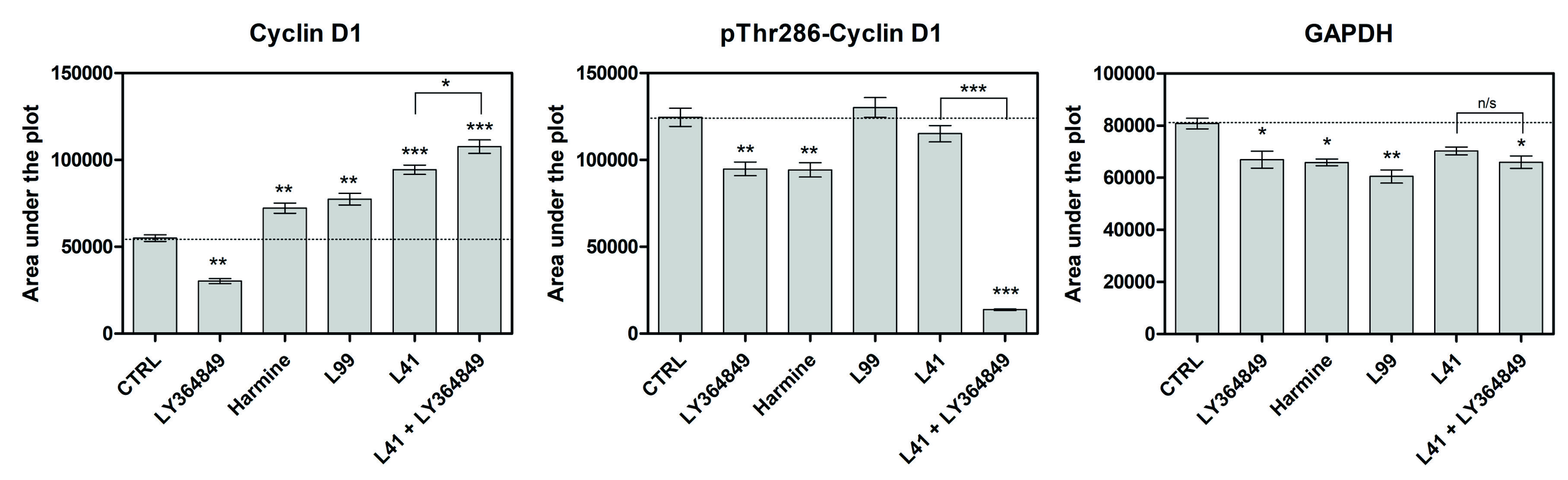

Supplement: S3 Fig — (TIF) [file pone.0285208.s003.tif]

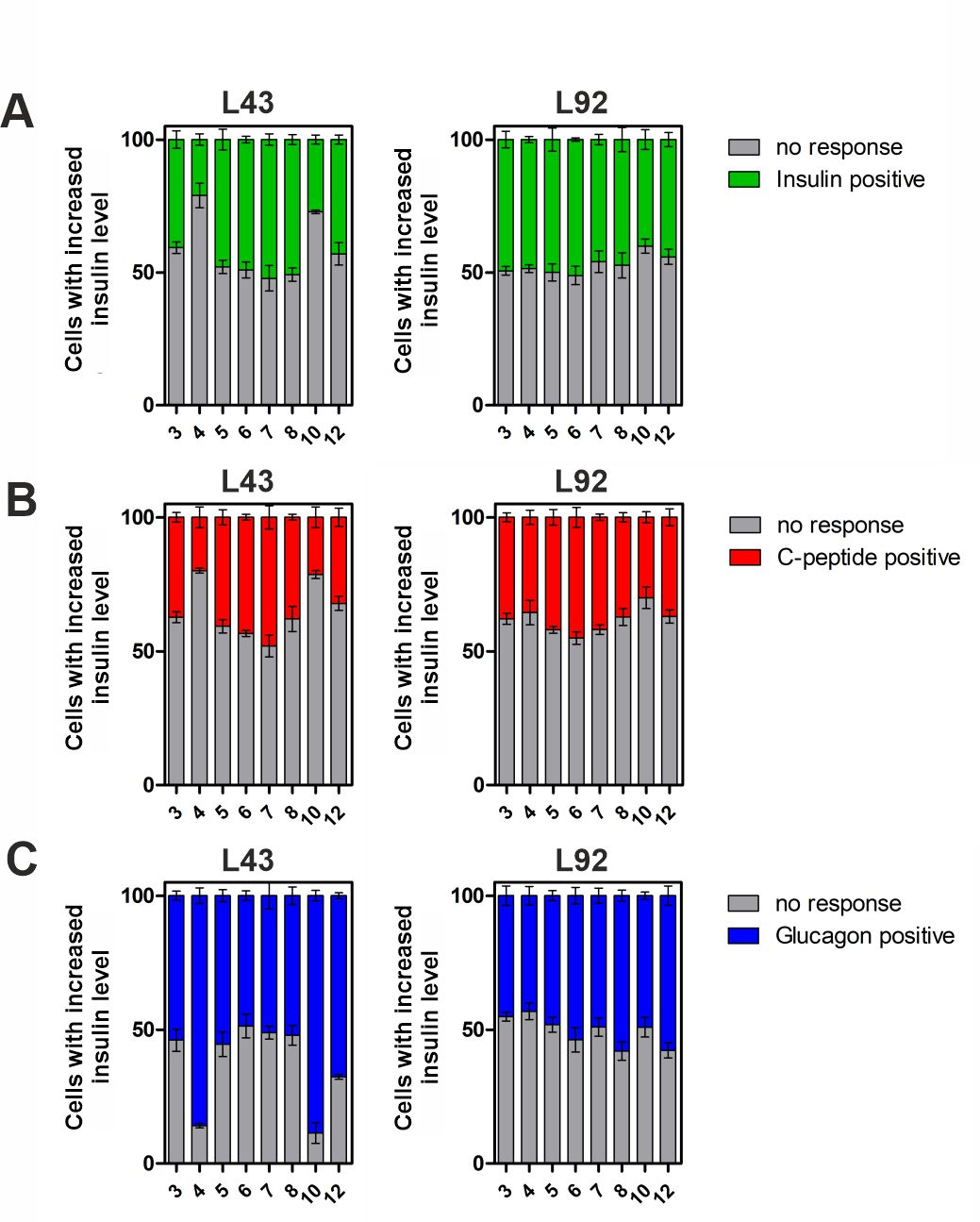

Supplement: S4 Fig — (TIF) [file pone.0285208.s004.tif]

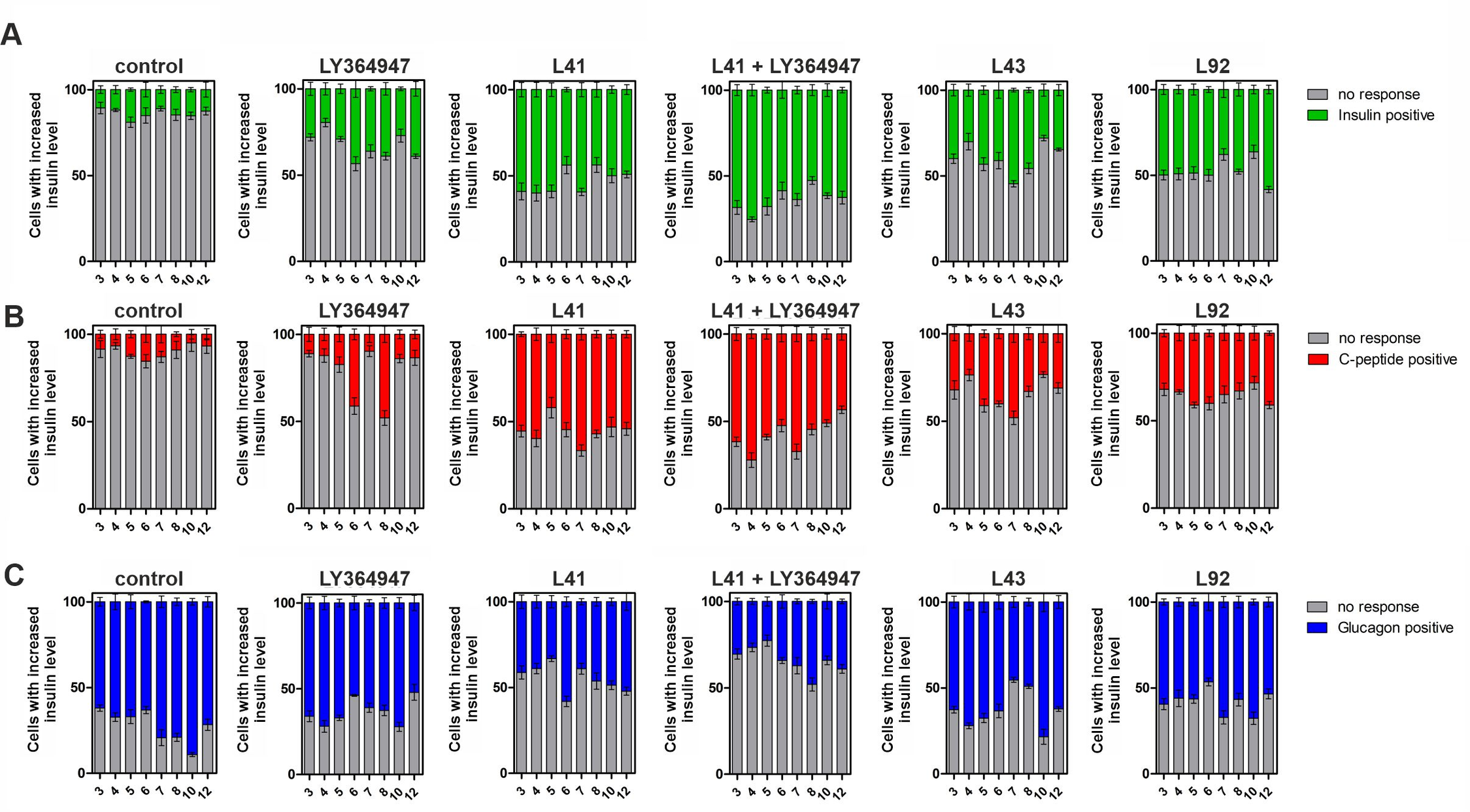

Supplement: S5 Fig — (TIF) [file pone.0285208.s005.tif]

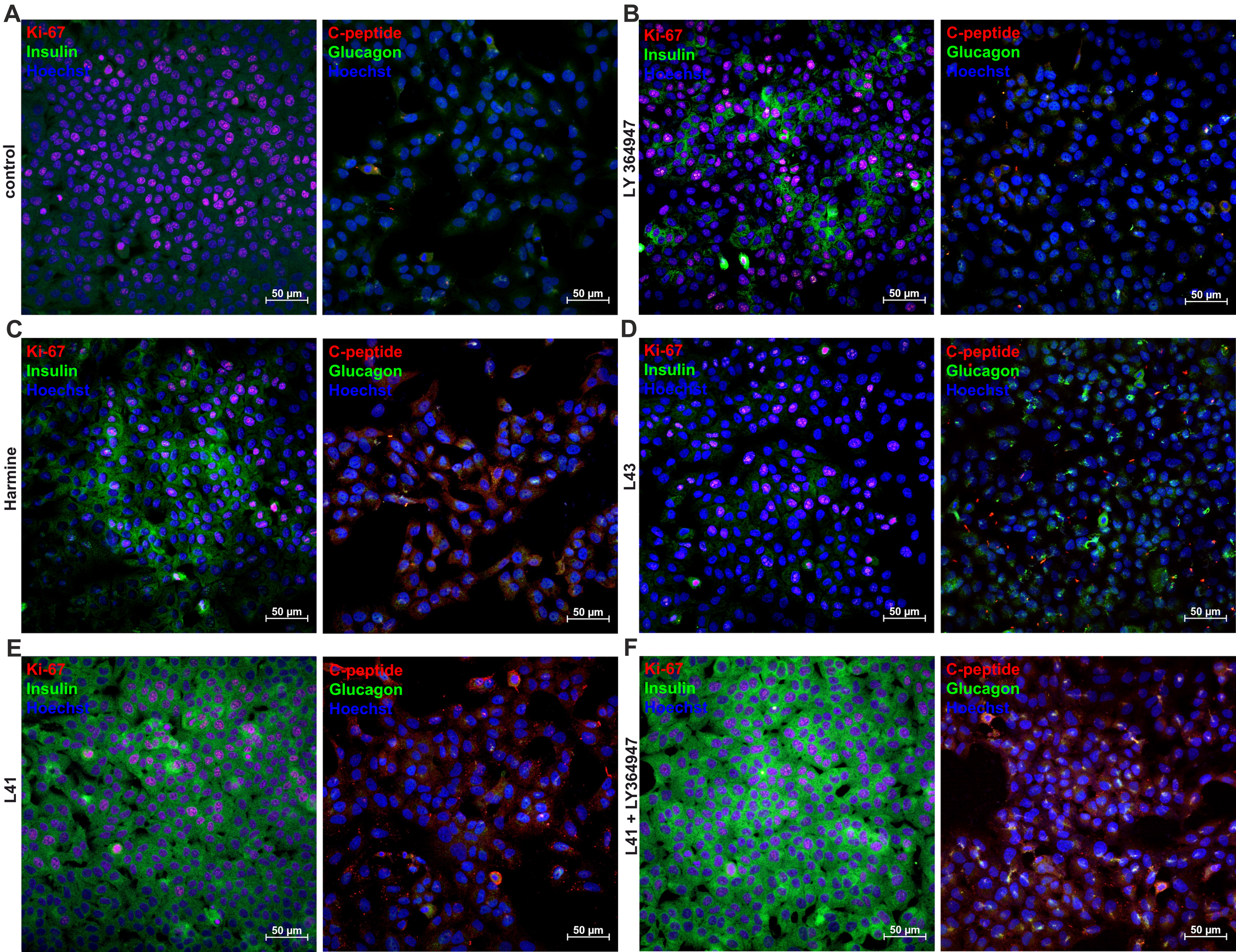

Supplement: S6 Fig — Proliferation rate (Ki67), insulin (INS) secretion and expression levels of C-peptide and glucagon. (TIF) [file pone.0285208.s006.tif]

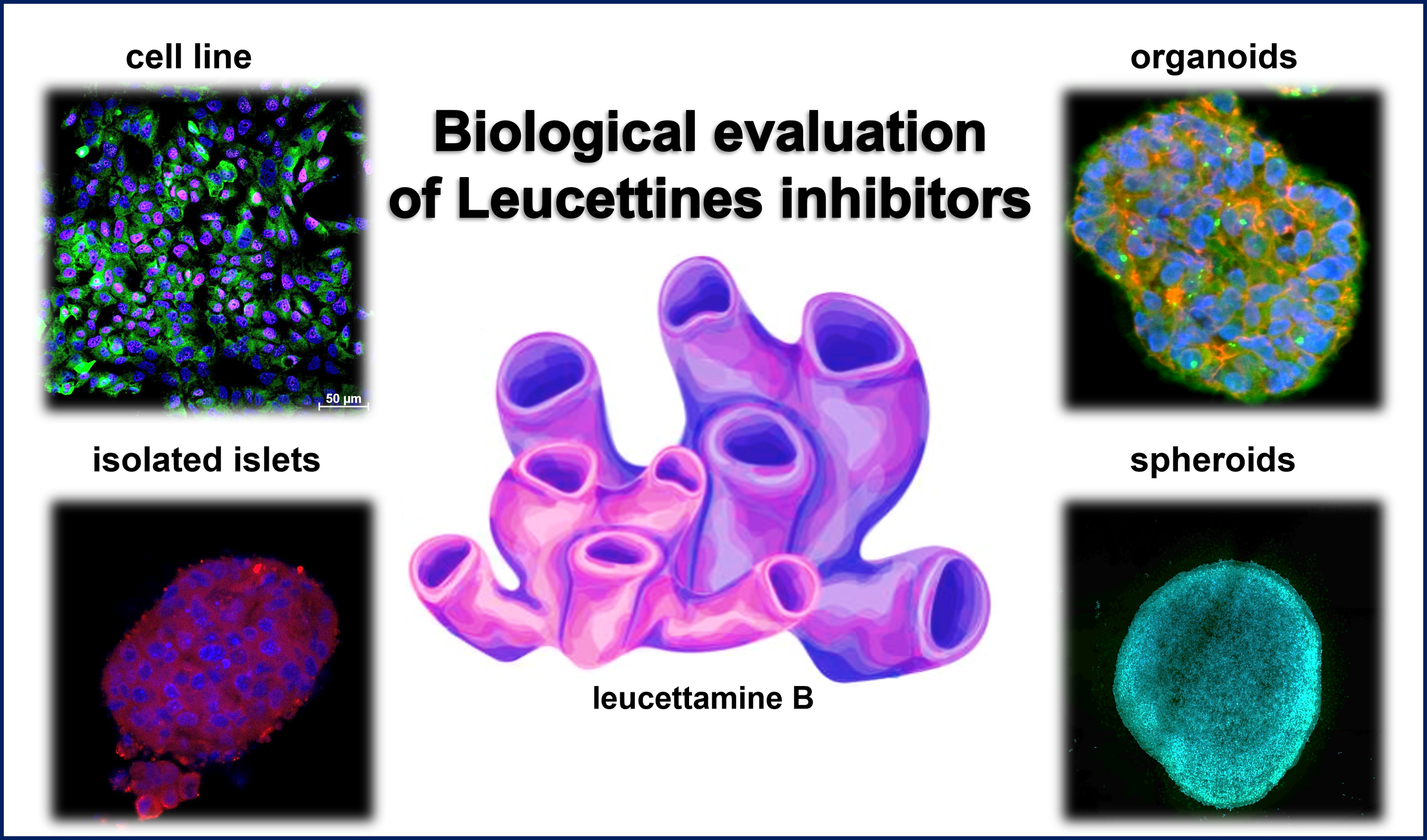

Supplement: S1 Graphical abstract — (TIF) [file pone.0285208.s007.tif]
